# Frailty and nursing care demand in hospitalized older adults: a cross-sectional study*

**DOI:** 10.1590/1518-8345.7747.4716

**Published:** 2025-11-10

**Authors:** Rosane Kraus, Maria Helena Lenardt, Clovis Cechinel, João Alberto Martins Rodrigues, Daiane Maria da Silva Marques, José Baudilio Belzarez Guedez

**Affiliations:** 1 Universidade Federal do Paraná, Curitiba, PR, Brazil. Universidade Federal do Paraná PR Curitiba Brazil

**Keywords:** Nursing Care, Hospitalization, Frailty, Nurses Improving Care for Health System Elders, Frail Elderly, Cross-Sectional Studies

## Abstract

to analyze the relation between nursing care demand and frailty condition and markers in hospitalized older adults.

analytical cross-sectional study with a sample of 400 older adults. Data collection used a sociodemographic questionnaire, frailty phenotype tests, and a nursing care complexity assessment scale. Fisher’s exact test, Kruskal-Wallis test and Dunn’s test were applied, considering statistical significance for *p*≤0.05.

there was a predominance of pre-frail (48.7%), followed by frail (35.8%) and non-frail individuals(15.5%). Most frail older adults required high-dependency nursing care (44.8%). The minimum nursing care demand was 80.6% for non-frail, 60.5% for pre-frail, and 14% for frail older adults. Frail older adults required higher nursing care level than the non-frail in the areas: mental state, oxygenation, motility, ambulation, elimination, and therapy (*p*<0.001). There was association between minimal to intensive nursing care demand and reduced hand grip strength (*p*<0.001), reduced gait speed (*p*<0.001), fatigue/exhaustion (*p*<0.001), reduced physical activity level (*p*<0.001), and unintentional weight loss (*p*<0.019).

higher nursing care demand was associated with worse frailty condition and markers. Frailty assessment is indispensable to trace specific needs and support nursing care planning.

## Highlights

**(1)** Nursing care levels vary between frailty conditions. **(2)** Frail older adults require higher nursing care level than the non-frail. **(3)** Nursing care demand is proportional to frailty condition and markers. **(4)** Frailty condition assessment at hospital admission is fundamental. **(5)** Ambulation, body care and eliminations require higher nursing care level among the frail.

## Introduction

As individuals age, they are subject to the occurence of multimorbidities, functional decline, sarcopenia, cognitive decline, malnutrition and frailty, which are factors associated with increased hospitalization rates^(1)^.

Frailty in older adults is a clinical state characterized by increased vulnerability, in addition to being one of the main factors associated with functional decline and early mortality when there is exposure to a stressor^(2)^. It is a predictive factor for functional dependency, resulting from reduced muscle strength, endurance and physical performance. Assessing frailty phenotype provides a classification of frailty condition as non-frail, pre-frail and frail, with the markers: reduced gait speed (GS), reduced hand grip strength (HGS), unintentional weight loss, reduced physical activity level, and self-reported fatigue/exhaustion^(3)^.

Assessing frailty phenotype markers is essential in the hospital setting, considering that frail persons have a higher risk of complications, not related to the initial reason for hospitalization^(4)^. Therefore, nursing interventions should be planned according to the specific demands arising from the frailty condition.

The identification of care needs is an essential requirement for planning nursing care, and it is recognized that the nursing care demand in hospitalized older adults is higher than that observed in other age groups^(5)^. The Patient Classification System (PCS) developed by Fugulin^(6)^ is widely used for its capacity to analyze multiple dimensions of nursing care, providing a systematic and comprehensive approach to identify specific needs that orient nursing care^(7)^.

The relation between nursing care demand and frailty condition and markers in hospitalized older adults is still little explored in national and international studies. The increasing prevalence of frailty with the aging process highlights the need to identify the nursing care demands and needs of this hospitalized population segment. This research contributes to the development and structuring of targeted interventions (via systematization of nursing care) and specific nursing care plans.

Thus, this study aimed to analyze the relation between nursing care demand and frailty condition and markers in hospitalized older adults.

## Method

For systematic construction of the study, we used the guidance tool called Strengthening the Reporting of Observational Studies in Epidemiology (STROBE)^(8)^.

### Study design and location

This is a cross-sectional and analytical study with older adults in a medium complexity hospital, a reference for older adults, located in the southern region of Brazil.

### Population, participants, inclusion and exclusion criteria

Older adults hospitalized in the ward took part in this study. Inclusion criteria: older than or equal to 60 years, being hospitalized in clinical or surgical wards, having the nursing care demand assessed during hospitalization and presenting cognitive capacity, screened by the Mini-Mental State Examination (MMSE)^(9)^, considering the education level for cutoff points^(10)^. Exclusion criteria: having an indication for admission to the Intensive Care Unit (ICU) and being under droplet precaution. Companion inclusion criteria: being aged 18 years or older and having been accompanying the elderly for at least three months; for companions aged 60 years or older, cognitive screening was performed using the MMSE^(9)^, with cutoff points adopted according to education level^(10)^.

### Sample calculation and data collection

Sample calculation was used for a population aged ≥ 60 years, representative of hospitalizations in 2019 (n=4,146). The 95% confidence level was set with a sampling error of 5%. Considering the values for each parameter, a minimum sample size of 350 individuals was obtained. In this study, 400 participants were adopted as the sample size in order to achieve a sampling error of less than 5%.

Prior to data collection, in order to standardize the execution and minimize collection biases, theoretical and practical training was carried out with the team of examiners, members of the research group. The team consisted of undergraduate and graduate students from a federal higher education institution. Data were obtained from March 2022 to March 2023. The collection instruments had an average application time of approximately 30 minutes.

Initially, cognitive screening adopted the MMSE^(9)^, validated for Brazilian Portuguese, and different cutoff points were defined according to the education level of the participants. For illiterate individuals, the cutoff point was set at 13 points or less. For those with education between one year and eight incomplete years, the adopted cutoff point was 18 points or less. For participants with eight years or more of education, the limit score was set at 26 points or less^(10)^. Participants with MMSE scores below the specific cutoff points for the education level were excluded because they indicated a possible cognitive deficit, with the objective of selecting older adults able to answer the study questionnaires and sign the Informed Consent Form (ICF).

When the older person presented cognitive alteration, the companion was invited to answer the questions in the presence of the older person and sign the ICF. In cases in which the companion at the time of the interview did not feel secure to answer the questions, the team waited for replacement with the main companion, with whom contact was resumed in person at a subsequent visit. This procedure aimed to ensure data reliability. The ICF was then signed by the interviewed companion.

The collection consisted of the application of a sociodemographic questionnaire that included: sex, age, skin color, education level and household income. The recommended frailty phenotype tests were applied for the following components: HGS and GS, fatigue/exhaustion, unintentional weight loss, and physical activity level. According to Fried’s criteria, older adults presenting three or more markers were considered frail; one or two, pre-frail; and none, non-frail^(3)^.

HGS was measured using a Jamar hydraulic dynamometer in kilogram/force (Kgf). The older adults were instructed to remain seated in a chair with their feet resting on the floor, elbow flexed at 90 degrees, with a firm arm against their torso and wrist in a neutral position. The dominant hand grip was adjusted to the dynamometer so the second phalanx of the second, third and fourth fingers touched the curve of the device’s handle. On command, the examinee performed three grips, interspersed with one minute for return of the force, and the average of the three measurements was considered. HGS values were adjusted according to the quartile of Body Mass Index (BMI) and sex, and those in the lowest quintile of strength were considered frail for this marker^(3)^.

For GS, the older adults were instructed to walk, in the usual way, on a flat surface, signaled by a measuring tape of 6.6 meters. Participants who depended on mobility or gait aids performed the test using these devices. When the older adults were bedridden, this component was assigned a value of zero.

The fatigue/exhaustion marker was identified by self-report. The older adults were asked about the frequency of the following situations in the last week: (A) they felt that they had to make an effort to manage to perform their daily tasks and (B) they felt that they were unable to carry out their activities. The answers were categorized into 0 – rarely or no part of the time (<1 day); 1 – a part or small part of the time (1-2 days); 2 – moderate amount of time (3-4 days); or 3 – most of the time. An answer “2” or “3” to any of the questions categorized the older adult as frail for this component^(3)^.

Unintentional weight loss was assessed using a platform and stage digital scale. It was measured by BMI, calculated from anthropometric measurements, associated with the self-reported answers of the older person to two questions: (A) “Have you lost weight in the last months?” and (B) “How many kilos?”.

Physical activity level was assessed using the Minnesotta Leisure Activity Questionnaire, validated for Brazilian older adults^(11)^. The questions related to the frequency and time of activities performed in the last two weeks. For the value in kilocalories, we multiplied the intensity of each activity in Metabolic Equivalent Tasks by the constant 0.0175 and the individual’s weight in kilograms, and the total value was divided by two to obtain the average energy expenditure per week. Following Fried’s criterion^(3)^, after adjusting for sex, older adults presenting values in the lowest quintile were classified as frail for this marker.

Nursing care demand was assessed using the Fugulin scale^(6)^, which analyzes 12 dimensions of nursing care: mental state, oxygenation, vital signs, motility, ambulation, feeding, body care, elimination, therapy, cutaneous and mucous integrity/tissue impairment, dressing, time used in dressing. Each dimension has four indicators, which are scored from one to four, according to nursing care dependency. The Fugulin scale has a final score ranging from 12 to 48 points, and the higher the score, the higher the nursing care need. The scores were used to classify nursing care need as follows: 12–17 points as minimal nursing care need; 18–22 points as intermediate nursing care need; 23–28 points as high-dependency nursing care need; 29–34 points as semi-intensive nursing care need; and 35–48 points as intensive nursing care need^(6)^.

### Data analysis and processing

A database was built in spreadsheets of Microsoft Excel 2016 software. The typing was performed with double checking of data. For descriptive analysis, we used means, medians, tables with frequency and ratios with their corresponding 95% confidence intervals (CI). The groups of frail, pre-frail and non-frail older adults^(3)^ were compared using Fisher’s exact test for categorical variables. For numerical and categorical variables, the comparison used the Kruskal-Wallis test, followed by the Dunn’s test for multiple comparisons. The statistical analyses adopted the significance level of *p*≤0.05.

### Ethical aspects

The study was approved by the Human Research Ethics Committee of the Health Sciences Sector of the Federal University of Paraná and by the Human Research Ethics Committee of the Municipal Health Department, opinion No.5.055.260. The ICF was signed by the older person and/or companion prior to the beginning of the research, in accordance with Resolution No. 466/2012. Data confidentiality was complied with as per Resolutions No. 510/2016, No. 580/2018 and other resolutions of the National Health Council in force in the period of this study.

## Results

Table 1 shows the results related to the sociodemographic characteristics of the 400 study participants. Of these, 55.8% (n=223) are women, 70.85% (n=283) self-declare as white, 36.5% (n=146) are aged over 80 years, 51.5% (n=206) have not completed elementary education, and 39.8% (n=159) earn 1 to 3 minimum monthly wages.

**Table 1-:** Frequency distribution of the sociodemographic characteristics of the older adults in the sample. Curitiba, PR, Brazil, 2024

**Characteristic**	n*=400	**(95% CI** ^†^ **)**
Sex		
Female	223	55.8% (50.7%–60.7%)
Male	177	44.3% (39.3%–49.3%)
Age		
60 to 69 years	118	29.5% (25.1%–34.3%)
70 to 79 years	136	34% (29.4%–38.9%)
80 years or more	146	36.5% (31.8%–41.5%)
Skin color		
Yellow	3	0.8% (0.19%–2.36%)
White	283	70.8% (66.0%–75.1%)
Mixed-race	90	22.6% (18.4%–28.31%)
Black	24	6% (3.96%–8.92%)
Education		
Illiterate	72	18% (14.4%–22.2%)
Literate	38	9.5% (6.89%–12.9%)
Incomplete middle school	206	51.5% (46.5%–56.5%)
Complete middle school	22	5.5% (3.56%–8.33%)
Incomplete high school	12	3% (1.63%–5.33%)
Complete high school	28	7% (4.78%–10.1%)
Incomplete higher education	4	1% (0.32%–2.72%)
Complete higher education	18	4.5% (2.77%–7.15%)
Household income (MW ^‡^ )		
No income	22	5.5% (3.56%–8.33%)
0-1	135	33.8% (29.2%–38.6%)
1.1-3	159	39.8% (35%–44.7%)
3.1-5	55	13.8% (10.6%–17.6%)
5.1-10	5	1.3% (0.46%–3.6%)
>10	1	0.3% (0.01%–1.61%)
Not informed	23	5.8% (3.76%–8.63%)

^*^n = Number of participants; ^†^CI = Confidence interval; ^‡^MW = Minimum wage in 2024: R$ 1,412.00^(12)^

Table 2 shows the predominance of pre-frail older adults, 48.7% (n=195); frail older adults reached 35.8% (n=143); and non-frail older adults totaled 15.5% (n=62). As for high-dependency nursing care need, the percentages for frail, pre-frail and non-frail older adults were as follows, respectively: 44.8% (n=64), 10.8% (n=21) and 3.2% (n=2), *p*<0.001. There was a statistically significant difference between the groups in relation to the minimum nursing care demand (*p*<0.001), indicating that the proportion of older adults who require minimum nursing care varies significantly according to frailty condition. The observed minimal nursing care demand was 80.6% for the non-frail, 60.5% for the pre-frail and 14% for the frail, suggesting that reduced frailty is associated with lower demand for more complex nursing care.

**Table 2-:** Association between frailty condition and nursing care demand for the older adults in the sample. Curitiba, PR, Brazil, 2024

**Categorization**	n*=400	**Frailty Classification**			*p* **-value** ^†^
		**Non-frail n=62**	**Pre-frail n=195**	**Frail n=143**	
**Minimum nursing care**	**188** **47%** **(42%−52%)**	**80.6%** **(68.3%−89.2%)**	**60.5%** **(53.3%−67.4%)**	**14%** **(8.96%−21%)**	**<0.001**
Intermediate nursing care	89 22.3% (18.3%−26.7%)	16.1% (8.41%−28.1%)	27.7% (21.7%−34.6%)	17.5% (11.8%−24.9%)	
**High-dependency nursing care**	**87** 21.6% (17.9%−26.2%)	3.2% (0.56%−12.2%)	10.8% (6.94%−16.2%)	**44.8%** **(36.5%−53.3%)**	
Semi-intensive nursing care	29 7.3% (4.99%−10.4%)	0% (0%−7.27%)	1% (0.18%−4.05%)	18.8% (13%−26.5%)	
Intensive nursing care	7 1.8% (0.77%−3.73%)	0% (0%−7.27%)	0% (0%−2.41%)	4.9% (2.16%−10.2%)	

^*^n = Number of participants; ^†^*p* = Probability of significance by Fisher’s exact test

Table 3 shows the association of the score for each nursing care area with the different frailty conditions. Older adults classified as frail (n=143) required higher nursing care level than the non-frail in the nursing care areas: mental state, oxygenation, motility, ambulation, elimination and therapy (*p*<0.001). When compared to the pre-frail, the frail had higher scores in all nursing care areas, tissue impairment (*p*=0.031), dressing (*p*=0.004) and time used to perform dressings (*p*<0.001). Pre-frail patients reached higher scores than non-frail patients in the areas of ambulation (*p*<0.001), body care (*p*<0.001), and elimination (*p*<0.001). When comparing the total Fugulin scores^(6)^, among the three groups of patients, all groups were different from one another, and the mean scores were higher with increasing frailty (*p*= 0.001).

**Table 3-:** Association between mean score for nursing care areas and frailty of the older adults in the sample. Curitiba, PR, Brazil, 2024

**Nursing care area**	**Fried Classification**			*p* **-value** ^†^	**Significant differences** ^‡^
	**Non-frail** n*=62	**Pre-frail** n*=195	**Frail** n*=143		
Mental state	1 (1−1.1)	1.1 (1.1−1.2)	1.8 (1.7−2)	<0.001	Frail – Non-Frail, Frail – Pre-Frail
Oxygenation	1.1 (1−1.2)	1.2 (1.2−1.3)	1.6 (1.5−1.8)	<0.001	Frail – Non-Frail, Frail – Pre-Frail
Vital signs	2	2	2	0.298	
Motility	1.2 (1.1−1.3)	1.4 (1.3−1.5)	2.4 (2.2−2.6)	<0.001	Frail – Non-Frail, Frail – Pre-Frail
Ambulation	1.1 (1−1.3)	1.6 (1.5−1.8)	3 (2.8−3.2)	<0.001	Frail – Non-Frail, Frail – Pre-Frail, Non-Frail – Pre-Frail
Eating	1 (1−1)	1.1 (1−1.1)	1.6 (1.5−1.8)	<0.001	Frail – Non-Frail, Frail – Pre-Frail
Body care	1.3 (1.1−1.4)	1.8 (1.6−2)	3.2 (3−3.4)	<0.001	Frail – Non-Frail, Frail – Pre-Frail, Non-Frail – Pre-Frail
Elimination	1.4 (1.2−1.6)	1.8 (1.6−1.9)	2.8 (2.7−3)	<0.001	Frail – Non-Frail, Frail – Pre-Frail, Non-Frail – Pre-Frail
Therapy	2 (2−2)	2 (2−2)	2.1 (2−2.2)	<0.001	Frail – Non-Frail, Frail – Pre-Frail
Tissue impairment	1.3 (1.1−1.4)	1.2 (1.1−1.3)	1.3 (1.2−1.5)	0.031	Frail – Pre-Frail
Dressing	1.1 (1−1.2)	1.1 (1−1.1)	1.2 (1.1−1.3)	0.004	Frail – Pre-Frail
Time for dressing	1.1 (1−1.2)	1 (1−1.1)	1.2 (1.1−1.3)	<0.001	Frail – Pre-Frail
Total score	15.6 (15.0−16.3)	17.3 (16.8−17.8)	24.4 (23.5−25.4)	<0.001	Frail – Non-Frail, Frail – Pre-Frail, Non-Frail – Pre-Frail

*n = Number of participants; ^†^*p* = Probability of significance by Kruskal-Wallis test; ^‡^Dunn’s test

Older adults under intensive nursing care showed reduced HGS, reduced GS, and self-reported fatigue/exhaustion (100%), and 85.7% had decreased physical activity level and unintentional weight loss. For older adults under minimal nursing care, 7.4% had reduced HGS, 3.2% had reduced gait speed, 25.5% had decreased physical activity level, 37.8% had unintentional weight loss, and 43.1% had self-reported fatigue/exhaustion. There is a statistically significant difference between the groups in relation to all frailty phenotype markers, as indicated by the *p*-values, showing association between higher nursing care need and worse frailty markers (Table 4).

**Table 4-:** Association between nursing care need and frailty phenotype markers for older adults in the sample. Curitiba, PR, Brazil, 2024

**Marker**	n*=400	**Nursing care level**					**p-value** ^†^
		**Minimum** n*=188	**Intermediary** n*=89	**High-dependency** n*=87	**Semi-intensive** n*=29	**Intensive** n*=7	
Reduced hand grip strength	23%	7.4% (4.29%–12.4%)	14.6% (8.31%–24%)	40.2% (30%–51.3%)	79.3% (59.7%–91.3%)	100% (56.1%–100%)	**<0.001**
Reduced gait speed	41%	3.2% (1.3%–7.14%)	41.6% (31.4%–52.5%)	96.6% (89.5%–99.1%)	100% (85.4%–100%)	100% (56.1%–100%)	**<0.001**
Decreased physical activity level	44%	25.5% (19.6%–32.5%)	41.6% (31.4%–52.5%)	71.3% (60.4%–80.2%)	82.8% (63.5%–93.5%)	85.7% (42%–99.2%)	**<0.001**
Unintentional weight loss	55%	37.8% (30.9%–45.1%)	40.4% (30.3%–51.4%)	49.4% (38.6%–60.3%)	58.6% (39.1%–75.9%)	85.7% (42%–99.2%)	**0.019**
Self-reported fatigue/exhaustion	43%	43.1% (36%–50.5%)	55.1% (44. 2%–65.5%)	65.5% (54.5%–75.2%)	82.8% (63.5%–93.5%)	100% (56.1%–100%)	**<0.001**

*n = Number of participants; ^†^*p* = Probability of significance by Fisher’s exact test

## Discussion

Of the 400 study participants, there was a predominance of women, white skin color, aged 80 years or over, incomplete elementary education and with a family income of 1 to 3 minimum monthly wages. The number of women in the hospital setting reflects the process of feminization of aging, representing 51.5% of the country’s elderly population^(13)^.

In this study, the representativeness of individuals older than 80 years was 36.5%. Similar data was found in the study conducted in São Paulo (Brazil), with 865 hospitalized older adults, which identified the female sex in 53.9% and the oldest old-adults 64.6%^(14)^. The largest portion of the participants had low education (incomplete elementary education), notably 18% of illiterates. This percentage is similar to the national index recorded in Brazil (16%)^(13)^.

The predominance of pre-frail (48.8%) followed by frail (35.8%) contrasts with that identified in a systematic review, which aimed to identify the prevalence of frailty in 467,779 hospitalized older adults, aged 65 years or older. The results showed 47% of frail and 26% of pre-frail. However, the authors indicate the imprecision in the operational definition of frailty in researches as a point to be discussed^(15)^.

In this study, approximately 50% of the older adults required minimal nursing care, and about 20% required intermediate and high-dependency nursing care. A retrospective study evaluated 161 medical records of older adults in a university hospital in Maranhão (Brazil). Of the sample, 63.4% required minimal nursing care, 15% intermediate nursing care, 14.2% high-dependency nursing care, 5.6% semi-intensive nursing care and 1.8% intensive nursing care^(7)^.

Another national study, conducted in the emergency room of a hospital, aimed to classify the degree of dependency of admitted patients. Of the 783 individuals evaluated, 40.8% were aged 60 years or older. It was observed that 37% needed minimal nursing care, 31.7% required intermediate nursing care, 22.8% needed high-dependency nursing care, 7.4% needed semi-intensive nursing care and 1.3% required intensive nursing care^(16)^. These results, when compared to this study, have the same classification sequence, with a 10% lower value for minimal nursing care and a 10% higher value for intermediate nursing care. These differences can be attributed to variations in the profile of the older adults, as well as to the different characteristics of the hospital settings.

The higher nursing care demand value is associated with higher frailty. Among the frail, respectively, the results for those who needed high-dependency, semi-intensive, and intensive nursing care were as follows: 44.8%, 18.9% and 4.9%. For pre-frail patients, 60.5% needed minimal nursing care; 27.7%, intermediate nursing care; and 10.8, high-dependency nursing care. In turn, for non-frail patients, 80.6% required minimal nursing care and 16.1%, intermediate nursing care. There was a significant gap of studies in the national and international literature on the relation between nursing care levels and frailty condition in hospitalized older adults. Thus, we note the cross-sectional study conducted in a Long-Stay Institution (LSI) in China. The data indicated association between frailty and care needs (OR 3.06, 95% CI; 2.06-4.55, *p*<0.01). According to the researchers, there is a linear relation between frailty and care needs. As the degree of frailty increases, the older adults’ long-term care needs increase drastically^(17)^.

Dissimilarities were identified among older adults in the non-frail, pre-frail and frail conditions in all areas evaluated. Frail older adults require higher nursing care levels and more interventions when compared to pre-frail older adults, as shown in the areas of mental state, oxygenation, motility, ambulation, eating, body care, elimination, therapy and tissue impairment. For frail older adults, we note disabilities as to motility (moving with assistance or unable to move), ambulation (restricted to bed or bedridden), body care (bed bath and body hygiene performed by the nursing team), elimination (use of bedpan or bed eliminations). These disabilities were considered the most limiting and those that showed the highest nursing care need (*p*<0.001). Frailty can be considered a pre-incapacitating factor^(2)^, which hinders basic activities of daily living, being associated with cognitive impairment, increased propensity to falls^(18)^, and increased susceptibility to risk of violence for older adults^(19)^.

As for frailty phenotype markers, prevalence increased as nursing care demand grew. The markers for reduced GS and HGS, self-reported fatigue/exhaustion were identified in 100% of older adults with intensive nursing care needs. There was association between their nursing care need and frailty phenotype markers.

Frailty syndrome is a physiological precursor to disability, characterized by weakness, decreased endurance, and slow performance. These central aspects of frailty mainly affect energy and speed-dependent functions, such as mobility^(3)^. A study with hospitalized older adults identified frailty as a predictor of decreased mobility^(20)^. Targeted interventions can halt, slow, or reverse this decline^(2)^. This study outcomes indicate that, with increasing nursing care need – from minimal to intensive –, there is a proportional increase in muscle weakness, as observed in greater functional limitations.

The predominance frailty markers, such as reduced GS, reduced HGS and self-reported fatigue/exhaustion, in older adults who required intensive nursing care, indicates a substantial loss of muscle strength and endurance, identified according to the nursing care need areas. These markers are indicative of probable sarcopenia, which is progressive and widespread loss of muscle mass and strength and/or function^(21)^. Sarcopenia and frailty are prevalent conditions in hospitalized older adults^(22)^.

Self-reported fatigue/exhaustion was the most prevalent marker among older adults under minimal and intermediate nursing care. Thus, the older adults’ self-report of this isolated marker anticipates in them the emergence of frailty. In studies, it is often predominant when compared to other markers. Data from a cross-sectional study in a hospital in Iran indicated the exhaustion marker present in 83% of hospitalized older adults^(23)^. In other cross-sectional study, pre-frail older adults without clinical signs of exhaustion experienced lower hand grip strength, increased self-perceived fatigue, and lower vitality levels compared to robust older adults (*p*<0.001)^(24)^.

Other marker with significant percentages was unintentional weight loss, self-reported in 43% of the hospitalized older adults. Frailty researchers advise health care professionals to trace the treatable causes of weight loss^(2)^. Individuals with unintentional weight loss (≥4.5 kg in one year) and individuals with unintentional loss of 25% of body weight over one year are considered the population at risk of nursing diagnosis – “risk of” or “frailty syndrome of the elderly”^(25)^. In the weight loss process, there is not only a reduction in adipose tissue, but also an accelerated decrease in lean mass, which is one of the main factors for the development of sarcopenia^(21)^. Notably, this variable corresponds to self-reported information, whose accuracy depends on the older adults’ preservation of cognitive functions and consciousness level.

Reduced GS reached 41% of the sample and was observed in 96.6% of the individuals with high-dependency nursing care needs and 100% in those in semi-intensive nursing care and with a less significant percentage in minimal nursing care (3.2%). Reduced gait speed affects nursing care areas such as: motility, ambulation, body care and elimination^(26)^. Frail older adults present worsened gait and performance of simultaneous tasks (motor and cognitive)^(27)^.

The reduced HGS frailty marker was the one that reached the lowest percentage (23%); however, it was present in all (100%) of the older adults in intensive nursing care. The manifestation of this marker seems to be related to the stimulus provided by the manual activities that the older adults still perform, even those who are bedridden. It corroborates the percentage found in a study in Spain that totaled a 20.4% reduction in HGS, and this marker was associated with lower probability of recovering non-frailty. The authors note that this frailty marker is often the last to manifest^(28)^. The more frequent use of the upper limbs in daily activities maintains the muscular strength and endurance of these limbs in older adults.

The frailty condition identified in the sample shows needs for nursing care in addition to care for the morbidity that motivated hospitalization. This additional care shows the importance of tracing the markers early and the need to incorporate frailty assessment into hospital admission^(29)^. Some studies found the need to improve the nurses’ skills in recognizing and managing the frailty condition^(30-31)^, as these professionals are in direct and frequent contact with hospitalized older adults^(32)^.

This approach enables directing nursing interventions geared toward promoting self-care, with an emphasis on strengthening functional skills, instead of focusing on the limitations of individuals^(33)^.

This study demonstrates that frailty compromises mobility, elimination, body care and therapy adherence. We note the importance of tracing this condition early in hospitalized older adults so as to support targeted interventions, optimize staff sizing, and enhance the health care plan, aiming to slow, halt or reverse the progression of frailty in the hospital setting. Health education for older adults and their families, combined with the strengthening of family support and the promotion of knowledge about the clinical condition and adherence to treatment, constitute a relevant strategy in the context of nursing care^(34)^.

The tracing of frailty is considered an initial care, but which provides direction for the care to be established, with the objective of avoiding the negative effects of the transition from frailty to more severe conditions during hospitalization. To this end, the nursing and multidisciplinary teams need to develop integrated health care plans, based on physical exercises to improve strength, endurance and aerobic fitness, nutrition with protein and caloric support, prescription of vitamin D supplementation, and reduction of polypharmacy^(2)^.

This study contributes to geriatric nursing by demonstrating the association between frailty markers (reduced hand grip strength, decreased gait speed, and fatigue) and increased functional dependency during hospitalization. The early tracing of these markers by nursing teams enables individualized interventions, favoring the maintenance of functionality and preventing adverse outcomes, such as immobility, delirium, falls and loss of autonomy.

In addition, the study contributes to the geriatric nursing clinical practice by proposing the incorporation of frailty assessment as a systematic tool at the time of hospital admission. This approach supports evidence-based nursing care planning and contributes to proper team sizing, rational resource allocation, and the development of more effective therapeutic plans. By recognizing frailty as a potentially reversible condition, this research also reinforces the importance of nursing care strategies focused on functional rehabilitation, health education and autonomy promotion, consistently with the principles of active aging and humanization of hospital care for older adults.

As a limitation, we note the use of the PCS instrument, which requires more comprehensive assessments, including social and psychological aspects, in addition to the scarcity of studies that relate frailty and nursing care demand, which restricted the discussion of the results. The use of self-reported data — such as on fatigue, exhaustion and weight loss — is also a limitation; however, this bias was minimized through methodological rigor, interviewer training, instrument calibration and statistical support. Observation bias was reduced with the development of a standardized collection and analysis protocol. As a positive point, we note the representativeness of the sample, which is from a hospital that is part of Brazil’s Unified Health System (SUS).

## Conclusion

The frailty condition is associated with increased nursing care demand in hospitalized older adults. It was found that frail older adults had a higher need for high-dependency care, while pre-frail and non-frail older adults predominantly required minimal nursing care. The most impaired areas among hospitalized frail older adults included ambulation, body care and elimination, showing functional limitations that require specific interventions of the nursing team.

In this context, we note the relevance of systematic frailty assessment at hospital admission, focusing on clinical markers such as reduced gait speed, reduced hand grip strength, and self-reported fatigue/exhaustion. The early tracing of these indicators can support individualized nursing care planning, optimize nursing team sizing, and favor the implementation of strategies aimed at maintaining or recovering functionality, thus contributing toward enhancing nursing care.

## Data Availability

Datasets related to this article will be available upon request to the corresponding author.
