# Supplementary material for: Frailty and nursing care demand in hospitalized older adults: a cross-sectional study
Source: Rev Lat Am Enfermagem. 2025 Nov 10;33:e4716. doi: 10.1590/1518-8345.7747.4716 (PMC12610926; doi:10.1590/1518-8345.7747.4716)
Supplement: Translation

# Translation: Fragilidade física e a demanda de cuidados em pessoas idosas hospitalizadas: estudo transversal*

**DOI:** 10.1590/1518-8345.7747.4717

**Published:** 2025

**Authors:** Rosane Kraus, Maria Helena Lenardt, Clovis Cechinel, João Alberto Martins Rodrigues, Daiane Maria da Silva Marques, José Baudilio Belzarez Guedez

**Affiliations:** 1 Universidade Federal do Paraná, Curitiba, PR, Brasil. Universidade Federal do Paraná PR Curitiba Brasil

**Keywords:** Cuidados de Enfermagem, Hospitalização, Fragilidade, Cuidado de Enfermagem ao Idoso Hospitalizado, Idoso Fragilizado, Estudos Transversais

## Abstract

analisar a relação entre demanda de cuidados de enfermagem e a condição e os marcadores de fragilidade física em pessoas idosas hospitalizadas.

estudo transversal analítico com amostra de 400 pessoas idosas. Para coleta de dados, empregou-se questionário sociodemográfico, testes do fenótipo de fragilidade física e escala de avaliação de complexidade assistencial. Aplicou-se o teste exato de Fisher, Kruskal-Wallis e teste de Dunn, considerando-se significância estatística para *p*≤0,05.

houve predomínio dos pré-frágeis (48,7%), seguidos dos frágeis (35,8%) e não frágeis (15,5%). A maioria dos frágeis exigiu cuidados de alta dependência (44,8%). A demanda de cuidados mínimos foi de 80,6% para os não frágeis, 60,5% para os pré-frágeis e 14% para os frágeis. Os frágeis demandaram assistência superior aos não frágeis nas áreas: estado mental, oxigenação, motilidade, deambulação, eliminação e terapêutica (*p*<0,001). Houve associação entre demanda de cuidado mínimo a intensivo e redução da força de preensão manual (*p*<0,001), redução da velocidade da marcha (*p*<0,001), fadiga/exaustão (*p*<0,001), redução do nível de atividade física (*p*<0,001) e perda de peso não intencional (p<0,019).

a maior demanda de cuidados de enfermagem associou-se à pior condição e aos marcadores de fragilidade física. A avaliação da fragilidade física é indispensável para identificar necessidades específicas e direcionar cuidados.

## Destaques

**(1)** Os níveis de cuidados de enfermagem variam entre as condições de fragilidade física. **(2)** Pessoas idosas frágeis demandam assistência de enfermagem superior aos não frágeis. **(3)** A necessidade de cuidados é proporcional à condição e aos marcadores de fragilidade. **(4)** Avaliação da condição de fragilidade física na admissão hospitalar é fundamental. **(5)** Deambulação, cuidado corporal e eliminações exigem maior cuidado entre os frágeis.

## Introdução

À medida que as pessoas envelhecem, verifica-se a ocorrência de multimorbidades, declínio funcional, sarcopenia, declínio cognitivo, desnutrição e fragilidade, fatores associados ao aumento das taxas de hospitalização^(1)^.

A fragilidade física na pessoa idosa é um estado clínico, caracterizado por aumento da vulnerabilidade, além de ser um dos principais fatores associados ao declínio funcional e à mortalidade precoce quando expostos a um estressor^(2)^. Constitui fator preditivo de dependência funcional, decorrente da redução da força muscular, resistência e desempenho físico. O fenótipo da fragilidade permite classificar a condição de fragilidade como não frágil, pré-frágil e frágil, mediante os marcadores: redução da velocidade da marcha (VM), redução da força de preensão manual (FPM), perda de peso não intencional, redução do nível de atividade física e autorrelato de fadiga/exaustão^(3)^.

A avaliação dos marcadores do fenótipo da fragilidade torna-se imprescindível no contexto hospitalar, tendo em vista que os frágeis apresentam maior risco de complicações, não relacionadas ao motivo inicial da hospitalização^(4)^. Portanto, as intervenções de enfermagem devem ser planejadas conforme as demandas específicas decorrentes da condição de fragilidade.

A identificação das necessidades de cuidados constitui requisito essencial para o planejamento da assistência de enfermagem, sendo reconhecido que a demanda por cuidados em idosos hospitalizados é superior à observada em outras faixas etárias^(5)^. O Sistema de Classificação de Pacientes (SCP) elaborado por Fugulin^(6)^ é amplamente utilizado pela capacidade de analisar múltiplas dimensões do cuidado de enfermagem, oferecendo uma abordagem sistemática e abrangente para identificar necessidades específicas que norteiam a assistência de enfermagem^(7)^.

A relação entre a demanda de cuidado e a condição e os marcadores de fragilidade física em pessoas idosas hospitalizadas ainda é pouco explorada em estudos nacionais e internacionais. A crescente prevalência da fragilidade física com o processo de envelhecimento ressalta a necessidade de identificar as demandas e necessidades de cuidados desse segmento populacional hospitalizado. Essa investigação contribui para a elaboração e estruturação de intervenções direcionadas (mediante sistematização da assistência) e de planos de cuidados específicos.

Considerando o exposto, este estudo teve como objetivo analisar a relação entre a demanda de cuidados de enfermagem e a condição e os marcadores de fragilidade física em pessoas idosas hospitalizadas.

## Método

Para a construção sistematizada do estudo, foi utilizada a ferramenta de orientação do *Strengthening the Reporting of Observational Studies in Epidemiology* (STROBE)^(8)^.

### Delineamento do estudo e local

Estudo de corte transversal e analítico, realizado com pessoas idosas em um hospital de média complexidade, referência para idosos, localizado na região Sul do Brasil.

### População, participantes, critérios de inclusão e exclusão

Pessoas idosas hospitalizadas em enfermaria constituíram a população do estudo. Os critérios de inclusão foram: ter idade maior ou igual a 60 anos, estar hospitalizado em enfermarias clínicas ou cirúrgicas, ter a demanda de cuidado avaliada durante a hospitalização e apresentar capacidade cognitiva, rastreada pelo Mini-Exame do Estado Mental (MEEM)^(9)^, considerando-se o nível de escolaridade para os pontos de corte^(10)^. Os critérios de exclusão foram: apresentar indicação de internamento em Unidade de Terapia Intensiva (UTI) e estar em precaução de gotículas. Os critérios de inclusão do acompanhante foram: ter idade igual ou superior a 18 anos e acompanhar o idoso há pelo menos três meses; para os acompanhantes com idade maior ou igual a 60 anos, foi realizado rastreio cognitivo por meio do MEEM^(9)^, com pontos de corte adotados segundo a escolaridade^(10)^.

### Cálculo da amostra e coleta de dados

Utilizou-se o cálculo amostral para população com idade ≥ 60 anos, representativa dos internamentos no ano de 2019 (n=4.146). Foram fixados o nível de confiança de 95% com erro amostral de 5%. Considerados os valores para cada parâmetro, obteve-se o tamanho amostral mínimo de 350 indivíduos. Neste estudo, adotou-se como tamanho da amostra 400 participantes, a fim de atingir um erro amostral inferior a 5%.

Previamente à coleta de dados, com o intuito de padronizar a execução e minimizar vieses de coleta, foram realizados treinamentos teóricos e práticos com a equipe de examinadores, membros do grupo de pesquisa. A equipe foi constituída por discentes de graduação e pós-graduação de uma instituição federal de ensino superior. Os dados foram obtidos a partir de março de 2022 até março de 2023. O tempo médio de aplicação dos instrumentos de coleta foi de aproximadamente 30 minutos.

Inicialmente, realizou-se o rastreio cognitivo mediante a aplicação do MEEM^(9)^, validado para o português do Brasil, e foram definidos os pontos de corte distintos conforme o nível de escolaridade dos participantes. Para indivíduos analfabetos, estabeleceu-se o ponto de corte em 13 pontos ou menos. Para aqueles com escolaridade entre um e oito anos incompletos, o ponto de corte adotado foi de 18 pontos ou menos. Já para os participantes com oito anos ou mais de escolaridade, o escore limite foi fixado em 26 pontos ou menos^(10)^. Foram excluídos os participantes cujos escores do MEEM ficaram abaixo dos pontos de corte específicos para o nível de escolaridade, por indicarem possível déficit cognitivo, com o objetivo de selecionar as pessoas idosas aptas para responder aos questionários do estudo e assinar o Termo de Consentimento Livre e Esclarecido (TCLE).

Quando a pessoa idosa apresentou alteração cognitiva, o acompanhante foi convidado a responder às questões na presença da pessoa idosa e assinar o TCLE. Nos casos em que o acompanhante, presente no momento da entrevista, não se sentisse seguro para responder às questões, a equipe aguardava a substituição pelo acompanhante principal, com quem o contato era retomado presencialmente em visita subsequente. Tal procedimento visava assegurar a fidedignidade dos dados. O TCLE era então assinado pelo acompanhante entrevistado.

A coleta consistiu na aplicação de questionário sociodemográfico que incluiu: sexo, idade, cor da pele, escolaridade e renda familiar. Os testes preconizados pelo fenótipo da fragilidade física foram aplicados aos seguintes componentes: FPM e VM, fadiga/exaustão, perda de peso não intencional e nível de atividade física. Seguindo os critérios de Fried, a pessoa idosa que apresentou três ou mais marcadores foi considerada frágil; um ou dois, pré-frágil; e nenhum, não frágil^(3)^.

A FPM aferiu-se por meio de dinamômetro hidráulico Jamar em quilograma/força (Kgf). A pessoa idosa foi orientada a permanecer sentada em uma cadeira com os pés apoiados no chão, cotovelo flexionado a 90 graus, com braço firme contra o tronco e punho em posição neutra. Ajustou-se a empunhadura da mão dominante ao dinamômetro, de modo que a segunda falange do segundo, terceiro e quarto dedos tocassem a curva da haste do dispositivo. Ao comando, o examinado realizou três preensões, intercaladas por um minuto para retorno da força, e considerou-se a média das três aferições. Os valores da FPM foram ajustados segundo o quartil do Índice de Massa Corpórea (IMC) e sexo, e aqueles que compreenderam o quintil mais baixo de força foram considerados frágeis para este marcador^(3)^.

Para a VM, a pessoa idosa foi orientada a caminhar, de maneira habitual, em superfície plana, sinalizada por fita métrica de 6,6 metros. Os participantes que dependiam de dispositivos de auxílio à mobilidade ou à marcha realizaram o teste com o uso desses equipamentos. Quando a pessoa idosa estava acamada, foi atribuído o valor zero para este componente.

O marcador fadiga/exaustão foi identificado por autorrelato. As pessoas idosas foram questionadas quanto à frequência das seguintes situações na última semana: (A) sentiu que teve que fazer esforço para dar conta de suas tarefas de todos os dias e (B) sentiu que não conseguiu levar adiante suas coisas. As respostas foram categorizadas em 0 – raramente ou nenhuma parte do tempo (<1 dia); 1 – uma parte ou pequena parte do tempo (1-2 dias); 2 – quantidade moderada de tempo (3-4 dias); ou 3 – na maioria das vezes. Uma resposta “2” ou “3” para qualquer uma das perguntas categorizou o idoso como frágil para este componente^(3)^.

Para avaliação da perda de peso não intencional, foi utilizada uma balança digital do tipo plataforma e estadiômetro. Mensurou-se pelo IMC, calculado a partir de medidas antropométricas, associada ao autorrelato do idoso em resposta a duas questões: (A) “O senhor(a) perdeu peso nos últimos meses?” e (B) “Quantos quilos?”.

O nível de atividade física foi avaliado pelo *Minnesotta Leisure Activity Questionnaire*, validado para idosos brasileiros^(11)^. Realizaram-se perguntas relacionadas à frequência e ao tempo de atividades realizadas nas últimas duas semanas. Para o valor em quilocalorias, utilizou-se a multiplicação da intensidade de cada atividade em *Metabolic Equivalent Tasks* pela constante 0,0175 e o peso do indivíduo em quilogramas, e o valor total foi dividido por dois para se obter o gasto energético médio por semana. Seguindo o critério de Fried^(3)^, após ajuste para sexo, a pessoa idosa que apresentou valores no menor quintil foi classificada como frágil para este marcador.

A demanda de cuidados foi avaliada pela da escala de Fugulin^(6)^, que analisa 12 dimensões do cuidado: estado mental, oxigenação, sinais vitais, motilidade, deambulação, alimentação, cuidado corporal, eliminação, terapêutica, integridade cutaneomucosa/comprometimento tecidual, curativo, tempo utilizado na realização de curativo. Cada dimensão apresenta quatro indicadores, que recebem pontuação de um a quatro, de acordo com a dependência da assistência de enfermagem. A graduação final da escala de Fugulin varia de 12 a 48 pontos, sendo que, quanto maior o escore, maior é a necessidade de assistência e cuidados de enfermagem. Foram considerados cuidados mínimos os escores de 12 a 17 pontos, cuidados intermediários os escores de 18 a 22 pontos, cuidados de alta dependência de 23 a 28 pontos, cuidados semi-intensivos de 29 a 34 pontos e cuidados intensivos acima de 34 pontos^(6)^.

### Análise e tratamento dos dados

Construiu-se um banco de dados em planilhas do *software Microsoft Excel* 2016. A digitação foi realizada com dupla checagem dos dados. Para a análise descritiva, utilizou-se médias, medianas, tabelas de frequência e proporções com seus respectivos intervalos de confiança (IC) de 95%. As comparações entre os grupos de pessoas idosas frágil, pré-frágil e não frágil^(3)^ foram realizadas pelo teste exato de Fisher, para variáveis categóricas. Para variáveis numéricas e categóricas, a comparação foi realizada pelo teste de Kruskal-Wallis, seguido do teste de Dunn para comparações múltiplas. Nas análises estatísticas, considerou-se o nível de significância de *p* ≤0,05.

### Aspectos éticos

O estudo foi aprovado pelo Comitê de Ética em Pesquisa em Seres Humanos do Setor de Ciências da Saúde da Universidade Federal do Paraná e pelo Comitê de Ética em Pesquisa em Seres Humanos da Secretaria Municipal de Saúde, parecer n^o^5.055.260. A assinatura do TCLE pela pessoa idosa e/ou acompanhante foi realizada previamente ao início da pesquisa, em conformidade com a Resolução nº 466/2012. Da mesma forma, respeitou-se o sigilo e a confidencialidade dos dados, nos termos expressos nas Resoluções nº** **510/2016, nº** **580/2018 e demais resoluções do Conselho Nacional de Saúde vigentes na data deste estudo.

## Resultados

Na Tabela 1, apresentam-se os resultados relativos às características sociodemográficas dos 400 participantes do estudo. Destes, 55,8% (n=223) são do sexo feminino, 70,85% (n=283) se autodeclaram brancos, 36,5% (n=146) têm idade acima de 80 anos, 51,5% (n=206) não concluíram o Ensino Fundamental e 39,8% (n=159) recebem de 1 até 3 salários mínimos.

**Tabela 1-:** Distribuição de frequência das características sociodemográficas das pessoas idosas da amostra. Curitiba, PR, Brasil, 2024

**Característica**	**n** * **=400**	**(IC** ^†^ **95%)**
Sexo		
Feminino	223	55,8% (50,7%-60,7%)
Masculino	177	44,3% (39,3%-49,3%)
Idade		
60 a 69 anos	118	29,5% (25,1%-34,3%)
70 a 79 anos	136	34% (29,4%-38,9%)
80 anos ou mais	146	36,5% (31,8%-41,5%)
Cor da pele		
Amarela	3	0,8% (0,19%-2,36%)
Branca	283	70,8% (66,0%-75,1%)
Parda	90	22,6% (18,4%-28,31%)
Preta	24	6% (3,96%-8,92%)
Escolaridade		
Analfabeto	72	18% (14,4%-22,2%)
Alfabetizado	38	9,5% (6,89%-12,9%)
Fundamental incompleto	206	51,5% (46,5%-56,5%)
Ensino Fundamental	22	5,5% (3,56%-8,33%)
Ensino Médio incompleto	12	3% (1,63%-5,33%)
Ensino Médio	28	7% (4,78%-10,1%)
Ensino Superior incompleto	4	1% (0,32%-2,72%)
Ensino Superior	18	4,5% (2,77%-7,15%)
Renda familiar (SM ^‡^ )		
Sem renda	22	5,5% (3,56%-8,33%)
0-1	135	33,8% (29,2%-38,6%)
1,1-3	159	39,8% (35%-44,7%)
3,1-5	55	13,8% (10,6%-17,6%)
5,1-10	5	1,3% (0,46%-3,6%)
Maior que 10	1	0,3% (0,01%-1,61%)
Não quer informar	23	5,8% (3,76%-8,63%)

^*^n = Número de participantes; ^†^IC = Intervalo de confiança; ^‡^SM = Salário-mínimo no ano de 2024: R$ 1.412,00^(12)^

Observa-se na Tabela 2 o predomínio de pessoas idosas pré-frágeis, 48,7% (n=195); os frágeis atingiram 35,8% (n=143); e os não frágeis totalizaram 15,5% (n=62). Em relação à necessidade de cuidados de alta dependência, as porcentagens para as pessoas idosas frágeis, pré-frágeis e não frágeis foram as seguintes, respectivamente: 44,8% (n=64), 10,8% (n=21) e 3,2% (n=2), *p*<0,001. Houve uma diferença estatisticamente significativa entre os grupos em relação à demanda por cuidados mínimos (*p*<0,001), indicando que a proporção de pessoas idosas que necessitam de cuidados mínimos varia de forma relevante conforme a condição de fragilidade física. A demanda por cuidados mínimos foi observada em 80,6% dos não frágeis, 60,5% dos pré-frágeis e 14% dos frágeis, sugerindo que a redução da fragilidade física está associada à menor demanda por cuidados mais complexos.

**Tabela 2-:** Associação entre condição de fragilidade física e demanda de cuidados das pessoas idosas da amostra. Curitiba, PR, Brasil, 2024

**Categorização**	n*=400	**Classificação Fragilidade**			**Valor *p*** ^†^
		**Não frágil** n=62	**Pré-frágil** n=195	**Frágil** n=143	
**Cuidados mínimos**	**188** **47%** **(42%-52%)**	**80,6%** **(68,3%-89,2%)**	**60,5%** **(53,3%-67,4%)**	**14%** **(8,96%-21%)**	**<0,001**
Cuidados intermediários	89 22,3% (18,3%-26,7%)	16,1% (8,41%-28,1%)	27,7% (21,7%-34,6%)	17,5% (11,8%-24,9%)	
**Cuidados de alta dependência**	**87** 21,6% (17,9%-26,2%)	3,2% (0,56%-12,2%)	10,8% (6,94%-16,2%)	**44,8%** **(36,5%-53,3%)**	
Cuidados semi-intensivos	29 7,3% (4,99%-10,4%)	0% (0%-7,27%)	1% (0,18%-4,05%)	18,8% (13%-26,5%)	
Cuidados intensivos	7 1,8% (0,77%-3,73%)	0% (0%-7,27%)	0% (0%-2,41%)	4,9% (2,16%-10,2%)	

^*^n = Número de participantes; ^†^*p* = Probabilidade de significância pelo teste exato de Fisher

Visualiza-se na Tabela 3 a associação da pontuação de cada área do cuidado às diferentes condições de fragilidade. As pessoas idosas classificadas como frágeis (n=143) exigiram assistência de enfermagem superior aos não frágeis nas áreas de cuidado: estado mental, oxigenação, motilidade, deambulação, eliminação e terapêutica (*p<*0,001). Quando comparados aos pré-frágeis, os frágeis tiveram maior pontuação em todas as áreas do cuidado, comprometimento tecidual (*p=*0,031), curativo (*p=*0,004) e tempo utilizado na realização dos curativos (*p<*0,001). Os pacientes pré-frágeis alcançaram pontuações maiores que os pacientes não frágeis nas áreas de deambulação (*p<*0,001), cuidado corporal (*p<*0,001) e eliminação (*p<*0,001). Quando comparadas as pontuações totais de Fugulin^(6)^, entre os três grupos de pacientes, todos os grupos foram diferentes entre si, e as pontuações médias foram maiores com o aumento da fragilidade (*p=*0,001).

**Tabela 3-:** Associação entre pontuação média das áreas de cuidado e fragilidade física das pessoas idosas da amostra. Curitiba, PR, Brasil, 2024

**Área do cuidado**	**Classificação de Fried**			**Valor *p*** ^†^	**Diferenças significativas** ^‡^
	**Não frágil** n*=62	**Pré-frágil** n*=195	**Frágil** n*=143		
Estado mental	1 (1-1,1)	1,1 (1,1-1,2)	1,8 (1,7-2)	<0,001	Frágil – Não Frágil, Frágil – Pré-Frágil
Oxigenação	1,1 (1-1,2)	1,2 (1,2-1,3)	1,6 (1,5-1,8)	<0,001	Frágil – Não Frágil, Frágil – Pré-Frágil
Sinais vitais	2	2	2	0,298	
Motilidade	1,2 (1,1-1,3)	1,4 (1,3-1,5)	2,4 (2,2-2,6)	<0,001	Frágil – Não Frágil, Frágil – Pré-Frágil
Deambulação	1,1 (1-1,3)	1,6 (1,5-1,8)	3 (2,8-3,2)	<0,001	Frágil – Não Frágil, Frágil – Pré-Frágil, Não Frágil – Pré-Frágil
Alimentação	1 (1-1)	1,1 (1-1,1)	1,6 (1,5-1,8)	<0,001	Frágil – Não Frágil, Frágil – Pré-Frágil
Cuidado corporal	1,3 (1,1-1,4)	1,8 (1,6-2)	3,2 (3-3,4)	<0,001	Frágil – Não Frágil, Frágil – Pré-Frágil, Não Frágil – Pré-Frágil
Eliminação	1,4 (1,2-1,6)	1,8 (1,6-1,9)	2,8 (2,7-3)	<0,001	Frágil – Não Frágil, Frágil – Pré-Frágil, Não Frágil – Pré-Frágil
Terapêutica	2 (2-2)	2 (2-2)	2,1 (2-2,2)	<0,001	Frágil – Não Frágil, Frágil – Pré-Frágil
Comprometimento tecidual	1,3 (1,1-1.4)	1,2 (1,1-1,3)	1,3 (1,2-1,5)	0,031	Frágil – Pré-Frágil
Curativo	1,1 (1-1,2)	1,1 (1-1,1)	1,2 (1,1-1,3)	0,004	Frágil – Pré-Frágil
Tempo para realização dos curativos	1,1 (1-1,2)	1 (1-1,1)	1,2 (1,1-1,3)	<0,001	Frágil – Pré-Frágil
Pontuação total	15,6 (15,0-16,3)	17,3 (16,8-17,8)	24,4 (23,5-25,4)	<0.001	Frágil – Não Frágil, Frágil – Pré-Frágil, Não Frágil – Pré-Frágil

*n = Número de participantes; ^†^*p* = Probabilidade de significância pelo teste de Kruskal-Wallis; ^‡^Teste de Dunn

Verifica-se que as pessoas idosas em cuidados intensivos apresentaram redução da FPM, da VM e autorrelato de fadiga/exaustão (100%), e 85,7% tiveram diminuição do nível de atividade física e perda de peso não intencional. Naquelas em cuidados mínimos, 7,4% foram identificadas com redução da FPM, 3,2% com redução da velocidade da marcha, 25,5%, tiveram diminuição do nível de atividade física, 37,8% apresentaram perda de peso não intencional e 43,1% relataram fadiga/exaustão. Verifica-se uma diferença estatisticamente significativa entre os grupos em relação a todos os marcadores do fenótipo de fragilidade, conforme indicado pelos valores de *p*, evidenciando associação entre maior necessidade de cuidados e pior desempenho nos marcadores de fragilidade (Tabela 4).

**Tabela 4-:** Associação entre demanda de cuidados e os marcadores do fenótipo de fragilidade das pessoas idosas da amostra. Curitiba, PR, Brasil, 2024

**Marcadores**	n*=400	**Nível de cuidados**					**Valor *p*** ^†^
		**Mínimos** n*=188	**Intermediários** n*=89	**Alta dependência** n*=87	**Semi-intensivos** n*=29	**Intensivos** n*=7	
Redução da força de preensão manual	23%	7,4% (4,29%-12,4%)	14,6% (8,31%-24%)	40,2% (30%-51,3%)	79,3% (59,7%-91,3%)	100% (56,1%-100%)	**<0,001**
Redução da velocidade da marcha	41%	3,2% (1,3%-7,14%)	41,6% (31,4%-52,5%)	96,6% (89,5%-99,1%)	100% (85,4%-100%)	100% (56,1%-100%)	**<0,001**
Diminuição do nível de atividade física	44%	25,5% (19,6%-32,5%)	41,6% (31,4%-52,5%)	71,3% (60,4%-80,2%)	82,8% (63,5%-93,5%)	85,7% (42%-99,2%)	**<0,001**
Perda de peso não intencional	55%	37,8% (30,9%-45,1%)	40,4% (30,3%-51,4%)	49,4% (38,6%-60,3%)	58,6% (39,1%-75,9%)	85,7% (42%-99,2%)	**0,019**
Autorrelato de fadiga/exaustão	43%	43,1% (36%-50,5%)	55,1% (44, 2%-65,5%)	65,5% (54,5%-75,2%)	82,8% (63,5%-93,5%)	100% (56,1%-100%)	**<0,001**

*n = Número de participantes; ^†^*p* = Probabilidade de significância pelo teste exato de Fisher

## Discussão

Dos 400 participantes do estudo, houve predomínio de mulheres, cor da pele branca, com idade igual ou superior a 80 anos, ensino fundamental incompleto e com renda familiar de 1 a 3 salários mínimos. O quantitativo de mulheres no cenário hospitalar reflete o processo de feminilização do envelhecimento, representando 51,5% da população idosa do país^(13)^.

Neste estudo, a representatividade de octogenários foi de 36,5%. Dado semelhante foi encontrado no estudo conduzido em São Paulo (Brasil), com 865 idosos hospitalizados, que identificou o sexo feminino em 53,9% e longevos 64,6%^(14)^. A maior parcela dos participantes apresentou baixa escolaridade (fundamental incompleto), notadamente 18% de analfabetos. Esse percentual é semelhante ao índice nacional registrado no Brasil (16%)^(13)^.

O predomínio de pré-frágeis (48,8%) seguido pelos frágeis (35,8%) contrasta com o identificado em revisão sistemática, a qual objetivou identificar a prevalência de fragilidade em 467.779 idosos hospitalizados, com idade maior ou igual a 65 anos. Os resultados apontaram 47% de frágeis e 26% de pré-frágeis. Entretanto, os autores indicam a imprecisão na definição operacional da fragilidade nas pesquisas como um ponto a ser discutido^(15)^.

Neste estudo, aproximadamente 50% das pessoas idosas necessitavam de cuidados mínimos, e cerca de 20%, de cuidados intermediários e de alta dependência. Um estudo retrospectivo avaliou 161 prontuários de idosos em um hospital universitário do Maranhão (Brasil). Da amostra, 63,4% necessitavam de cuidados mínimos, 15% cuidados intermediários, 14,2% alta dependência, 5,6% cuidados semi-intensivos e 1,8% cuidados intensivos^(7)^.

Outro estudo nacional, conduzido na emergência de um hospital, objetivou classificar o grau de dependência dos admitidos. Dos 783 indivíduos avaliados, 40,8% apresentavam idade maior ou igual a 60 anos. Observou-se que 37% precisavam de cuidados mínimos, 31,7% exigiam suporte intermediário, 22,8% necessitavam de cuidados de alta dependência, 7,4% tinham necessidade de uma assistência semi-intensiva e 1,3% requeriam cuidados intensivos^(16)^. Esses resultados, quando comparados aos deste estudo, apresentam a mesma sequência de classificação, com diminuição de 10% em cuidados mínimos e aumento de 10% em cuidados intermediários. Essas diferenças podem ser atribuídas às variações no perfil das pessoas idosas, bem como às distintas características dos ambientes hospitalares.

O maior quantitativo por demanda de cuidados de enfermagem está associado à maior fragilidade. Dos frágeis, respectivamente, os resultados dos que apresentaram cuidados de alta dependência, semi-intensivos e intensivos foram os seguintes: 44,8%, 18,9% e 4,9%. Dos pré-frágeis 60,5% apresentaram cuidados mínimos; 27,7%, cuidados intermediários; e 10,8, cuidados de alta dependência. Por sua vez, dos não frágeis, 80,6% demandavam cuidados mínimos e 16,1%, cuidados intermediários. Constatou-se expressiva lacuna de estudos na literatura nacional e internacional sobre a relação entre os níveis de cuidados de enfermagem e a condição de fragilidade física em pessoas idosas hospitalizadas. Sendo assim, destaca-se o estudo transversal que foi conduzido em uma Instituição de Longa Permanência (ILPI), na China. Os dados apontaram para associação entre fragilidade e as necessidades de cuidados (OR 3,06, IC 95%; 2,06-4,55, *p*<0,01). Para os pesquisadores, existe relação linear entre fragilidade e necessidades de cuidados. Conforme o crescente grau de fragilidade, as necessidades de cuidados de longa duração dos idosos aumentam drasticamente^(17)^.

Foram identificadas dessemelhanças entre as pessoas idosas nas condições não frágeis, pré-frágeis e frágeis em todas as áreas avaliadas. Pessoas idosas frágeis exigem maiores cuidados e intervenções de enfermagem quando comparados aos pré-frágeis, expressados nas áreas estado mental, oxigenação, motilidade, deambulação, alimentação, cuidado corporal, eliminação, terapêutica e comprometimento tecidual. Nas pessoas idosas frágeis, destacaram-se as incapacidades de motilidade (movimentação com auxílio ou incapaz de se movimentar), deambulação (restritos ao leito ou acamados), cuidado corporal (banho no leito e higiene corporal realizados pela equipe de enfermagem), eliminação (uso de comadre ou eliminações no leito). Essas incapacidades foram consideradas como as mais limitantes e as que expressavam maior necessidade de cuidados da enfermagem (*p*<0,001). A fragilidade física pode ser considerada como fator pré-incapacitante^(2)^, que dificulta a realização de atividades básicas de vida diária, está associada ao comprometimento cognitivo, aumento da propensão a quedas^(18)^, além de elevar a susceptibilidade ao risco de a pessoa idosa sofrer violência^(19)^.

Em relação aos marcadores do fenótipo da fragilidade física, houve aumento na prevalência à medida que crescia a demanda de cuidados. Os marcadores redução VM e FPM, autorrelato de fadiga/exaustão foram identificados em 100% das pessoas idosas com necessidades de cuidados intensivos. Houve associação entre os cuidados requeridos por elas e os marcadores do fenótipo de fragilidade física.

A síndrome da fragilidade é um precursor fisiológico da incapacidade, caracterizada por fraqueza, diminuição da resistência e desempenho lento. Esses aspectos centrais da fragilidade afetam, principalmente, as funções dependentes de energia e velocidade, como a mobilidade^(3)^. Um estudo com pessoas idosas hospitalizadas identificou a fragilidade como fator preditor de diminuição de mobilidade^(20)^. Intervenções direcionadas podem interromper, desacelerar ou reverter esse declínio^(2)^. Os resultados deste estudo indicam que, com o aumento da necessidade de cuidados – de mínimos a intensivos –, observa-se um incremento proporcional na fraqueza muscular, evidenciado por maiores limitações funcionais.

O predomínio dos marcadores de fragilidade física, como a redução da VM, a redução da FPM e o autorrelato de fadiga/exaustão, nas pessoas idosas que demandaram cuidados intensivos, indica uma perda substancial de força muscular e resistência, identificada pelas áreas de necessidade de cuidados. Esses marcadores são indicativos de provável sarcopenia, que é a perda progressiva e generalizada de massa e força e/ou função muscular^(21)^. A sarcopenia e a fragilidade física são condições prevalentes em idosos hospitalizados^(22)^.

O autorrelato de fadiga/exaustão foi o marcador mais prevalente entre as pessoas idosas em cuidados mínimos e intermediários. Desse modo, a referência pelas pessoas idosas desse marcador isolado antecipa nelas o surgimento da fragilidade física. Em estudos, frequentemente ele se mostra predominante, quando comparado aos outros marcadores. Dados de estudo transversal desenvolvido em um hospital do Irã apontaram o marcador exaustão presente em 83% dos idosos hospitalizados^(23)^. Em outro estudo transversal, os idosos pré-frágeis sem sinais clínicos de exaustão experimentavam menor força de preensão, maior fadiga autopercebida e níveis mais baixos de vitalidade em comparação aos robustos (*p*<0,001)^(24)^.

Outro marcador com percentuais expressivos foi a perda de peso não intencional, autorrelatada em 43% das pessoas idosas hospitalizadas. Estudiosos em fragilidade física aconselham os profissionais da área da saúde a identificarem as causas tratáveis de perda de peso^(2)^. Indivíduos com perda de peso não intencional (≥4,5 kg em um ano) e indivíduos com perda não intencional de 25% do peso corporal, ao longo de um ano, são considerados a população em risco do diagnóstico de enfermagem – “risco de” ou “síndrome da fragilidade do idoso”^(25)^. No processo de perda de peso, ocorre não apenas a redução do tecido adiposo, mas também a diminuição acelerada da massa magra, sendo este um dos principais fatores para o desenvolvimento da sarcopenia^(21)^. Ressalta-se que esta variável corresponde a uma informação autorreferida, cuja acurácia depende da preservação das funções cognitivas e do nível de consciência das pessoas idosas.

A redução da VM atingiu 41% da amostra e foi observada em 96,6% dos indivíduos com necessidades de cuidados de alta dependência e 100% naqueles em cuidados semi-intensivos e com percentual menos expressivo em cuidados mínimos (3,2%). A velocidade da marcha diminuída afeta as áreas de cuidado como: motilidade, deambulação, cuidado corporal e eliminação^(26)^. Idosos frágeis apresentam piora na marcha e no desempenho de tarefas simultâneas (motoras e cognitivas)^(27)^.

O marcador de fragilidade redução da FPM foi o que atingiu menor percentual (23%), no entanto, esteve presente na totalidade (100%) das pessoas idosas em cuidados intensivos. A manifestação deste marcador parece ter relação com o estímulo dado pelas atividades manuais que os idosos ainda realizam, até mesmo naqueles acamados. Corrobora o percentual aquele encontrado no estudo realizado na Espanha, que totalizou 20,4% de redução da FPM, e este marcador associou-se à menor probabilidade de recuperar a não fragilidade. Os autores acrescentam que tal marcador de fragilidade, frequentemente, é o último a se manifestar^(28)^. O uso mais frequente dos membros superiores em atividades diárias mantém a força e resistência muscular desses membros em pessoas idosas.

A condição de fragilidade física identificada na amostra expressa necessidades de cuidados de enfermagem em adição aos cuidados das morbidades, as quais motivaram a hospitalização. Nesse acréscimo aos cuidados, destaca-se a importância da identificação precoce dos marcadores e a necessidade de incorporar a avaliação da fragilidade na admissão hospitalar^(29)^. Alguns estudos identificaram a necessidade de aprimorar as habilidades dos enfermeiros no reconhecimento e manejo da condição de fragilidade^(30-31)^, por serem os profissionais que estão em contato direto e frequente com a pessoa idosa hospitalizada^(32)^.

Essa abordagem permite direcionar intervenções de enfermagem voltadas para a promoção do autocuidado, com ênfase no fortalecimento das habilidades funcionais, em vez de concentrar-se nas limitações dos indivíduos^(33)^.

Este estudo demonstra que a fragilidade física compromete a mobilidade, a eliminação, o cuidado corporal e a adesão terapêutica. Ressalta-se a importância da identificação precoce dessa condição em idosos hospitalizados para subsidiar intervenções direcionadas, otimizar o dimensionamento da equipe e qualificar o plano de cuidados, visando desacelerar, interromper ou reverter a progressão da fragilidade no contexto hospitalar. A educação em saúde direcionada à pessoa idosa e à família, aliada ao fortalecimento do apoio familiar e à promoção do conhecimento sobre a condição clínica e adesão ao tratamento, configura-se como estratégia relevante no âmbito do cuidado de enfermagem^(34)^.

Considera-se como um cuidado inicial, mas que fornece direção para os cuidados a serem estabelecidos, com os objetivos de se evitar os efeitos negativos da transição da fragilidade para condições superiores, no período de hospitalização. Para tanto, é necessário que a equipe de enfermagem e a equipe multiprofissional elaborem planos de cuidados integrados, baseados na prática de exercícios físicos de força, resistência e aeróbicos, nutrição com suporte proteico e calórico, prescrições de suplementação de vitamina D e redução da polifarmácia^(2)^.

Este estudo contribui para a enfermagem geriátrica ao demonstrar a associação entre marcadores de fragilidade (redução da força de preensão manual, diminuição da velocidade da marcha e fadiga) e o aumento da dependência funcional durante a hospitalização. A identificação precoce desses marcadores pela equipe de enfermagem possibilita intervenções individualizadas, favorecendo a manutenção da funcionalidade e prevenindo desfechos adversos, como imobilidade, delirium, quedas e perda de autonomia.

Além disso, o estudo subsidia a prática clínica da enfermagem geriátrica ao propor a incorporação da avaliação da fragilidade física como ferramenta sistemática no momento da admissão hospitalar. Essa abordagem subsidia o planejamento do cuidado com base em evidências e contribui para o dimensionamento adequado da equipe, alocação racional de recursos e elaboração de planos terapêuticos mais efetivos. Ao reconhecer a fragilidade como uma condição potencialmente reversível, esta pesquisa também reforça a importância de estratégias de cuidado centradas na reabilitação funcional, educação em saúde e promoção da autonomia, alinhando-se aos princípios do envelhecimento ativo e da humanização do cuidado hospitalar à pessoa idosa.

Como limitação, destaca-se o uso do instrumento SCP, que requer avaliações mais abrangentes, incluindo aspectos sociais e psicológicos, além da escassez de estudos que relacionem fragilidade física e demanda de cuidados, o que restringiu a discussão dos resultados. A utilização de dados autorreferidos, como fadiga, exaustão e perda de peso, também constitui limitação, embora esse viés tenha sido minimizado por meio de rigor metodológico, treinamento dos entrevistadores, calibração dos instrumentos e apoio estatístico. O viés de observação foi reduzido com o desenvolvimento de um protocolo padronizado de coleta e análise. Como ponto positivo, destaca-se a representatividade da amostra, proveniente de hospital vinculado ao Sistema Único de Saúde (SUS).

## Conclusão

A condição de fragilidade física está associada ao aumento da demanda de cuidados de enfermagem em pessoas idosas hospitalizadas. Verificou-se que idosos frágeis apresentaram maior necessidade de cuidados de alta dependência, enquanto os pré-frágeis e não frágeis demandaram predominantemente cuidados mínimos. As áreas de maior comprometimento entre os idosos frágeis hospitalizados incluíram deambulação, cuidado corporal e eliminação, evidenciando limitações funcionais que requerem intervenções específicas da equipe de enfermagem.

Nesse contexto, destaca-se a relevância da avaliação sistemática da fragilidade física na admissão hospitalar, com foco nos marcadores clínicos como a redução da velocidade da marcha e da força de preensão manual e o autorrelato de fadiga/exaustão. A identificação precoce desses indicadores pode subsidiar o planejamento individualizado da assistência, otimizar o dimensionamento da força de trabalho e favorecer a implementação de estratégias direcionadas à manutenção ou recuperação da funcionalidade, contribuindo para a qualificação do cuidado.

## Data Availability

Os conjuntos de dados relacionados a este artigo estarão disponíveis mediante solicitação ao autor correspondente.

# Translation: Fragilidad física y demanda de cuidados en adultos mayores hospitalizados: un estudio transversal*

**DOI:** 10.1590/1518-8345.7747.4715

**Published:** 2025

**Authors:** Rosane Kraus, Maria Helena Lenardt, Clovis Cechinel, João Alberto Martins Rodrigues, Daiane Maria da Silva Marques, José Baudilio Belzarez Guedez

**Affiliations:** 1 Universidade Federal do Paraná, Curitiba, PR, Brasil. Universidade Federal do Paraná PR Curitiba Brasil

**Keywords:** Atención de Enfermería, Hospitalización, Fragilidad, Nurses Improving Care for Health System Elders, Anciano Frágil, Estudios Transversales

## Abstract

analizar la relación entre demanda de cuidados de enfermería y la condición y los marcadores de fragilidad física en adultos mayores hospitalizados.

estudio transversal analítico con una muestra de 400 adultos mayores. Para la recopilación de datos, se utilizó un cuestionario sociodemográfico, pruebas de fenotipo de fragilidad física y una escala de evaluación de la complejidad asistencial. Se aplicaron las pruebas exacta de Fisher, Kruskal-Wallis y Dunn, considerando una significancia estadística para *p*≤0,05.

hubo un predominio de los prefrágiles (48,7%), seguidos de los frágiles (35,8%) y no frágiles (15,5%). La mayoría de los frágiles requirió cuidados de alta dependencia (44,8%). La demanda de cuidados mínimos fue del 80,6% para los no frágiles, del 60,5% para los prefrágiles y del 14% para los frágiles. Los frágiles requirieron asistencia superior que los no frágiles en los factores: estado mental, oxigenación, motilidad, deambulación, eliminación y terapéutica (*p*<0,001). Se observó una asociación entre la demanda de cuidados mínimos a intensivos y la reducción de la fuerza de agarre manual (*p*<0,001), reducción en la velocidad de la marcha (*p*<0,001), fatiga/agotamiento autoinformados (*p*<0,001), reducción del nivel de actividad física (*p*<0,001) y pérdida de peso no intencionada (*p*<0,019).

la mayor demanda de cuidados de enfermería se asoció con la peor condición y con los marcadores de fragilidad física. Evaluar la fragilidad física es esencial para identificar necesidades específicas y dirigir los cuidados.

## Destacados

**(1)** Los niveles de cuidados de enfermería varían según las condiciones de fragilidad física. **(2)** Los adultos mayores frágiles demandan asistencia de enfermería superior a los no frágiles. **(3)** La necesidad de cuidados es proporcional a la condición y a los marcadores de fragilidad. **(4)** La evaluación de la condición de fragilidad física al ingreso hospitalario es esencial. **(5)** La deambulación, el cuidado corporal y las eliminaciones requieren mayor cuidado entre los frágiles.

## Introducción

A medida que las personas envejecen, se observa la aparición de multimorbilidades, deterioro funcional, sarcopenia, deterioro cognitivo, desnutrición y fragilidad, factores asociados con el aumento de las tasas de hospitalización^(1)^.

La fragilidad física en el adulto mayor es un estado clínico que se caracteriza por una mayor vulnerabilidad, además de ser uno de los principales factores asociados al deterioro funcional y a la mortalidad temprana cuando se exponen a un estresor^(2)^. Es un factor predictivo de dependencia funcional, resultante de la reducción de la fuerza muscular, la resistencia y el rendimiento físico. El fenotipo de la fragilidad permite clasificar dicha condición como no frágil, prefrágil y frágil, mediante los marcadores: reducción de la velocidad de la marcha (VM), reducción de la fuerza de prensión manual (FPM), pérdida de peso no intencionada, reducción del nivel de actividad física y fatiga/agotamiento autoinformados^(3)^.

La evaluación de los marcadores del fenotipo de fragilidad se vuelve imprescindible en el contexto hospitalario, teniendo en cuenta que las personas frágiles presentan un mayor riesgo de complicaciones no relacionadas con el motivo inicial de la hospitalización^(4)^. Por lo tanto, las intervenciones de enfermería deben planificarse según las demandas específicas derivadas de la condición de fragilidad.

La identificación de las necesidades de cuidados es un requisito esencial para la planificación de la asistencia de enfermería, ya que se reconoce que la demanda de cuidados en adultos mayores hospitalizados es mayor que la observada en otros rangos de edad^(5)^. El Sistema de Clasificación de Pacientes (SCP) desarrollado por Fugulin^(6)^ es ampliamente utilizado por su capacidad de analizar múltiples dimensiones del cuidado de enfermería, ofreciendo un enfoque sistemático e integral para identificar necesidades específicas que guían la asistencia de enfermería^(7)^.

La relación entre la demanda de cuidados y la condición y los marcadores de fragilidad física en adultos mayores hospitalizados aún es poco explorada en estudios nacionales e internacionales. La creciente prevalencia de fragilidad física con el proceso de envejecimiento resalta la necesidad de identificar las demandas y necesidades de cuidados de este segmento poblacional hospitalizado. Esta investigación contribuye a la elaboración y estructuración de intervenciones dirigidas (mediante la sistematización de la asistencia) y de planes de cuidados específicos.

Considerando lo anterior, este estudio tuvo como objetivo analizar la relación entre la demanda de cuidados de enfermería y la condición y los marcadores de fragilidad física en adultos mayores hospitalizados.

## Método

Para la construcción sistematizada del estudio, se utilizó la herramienta de orientación *Strengthening the Reporting of Observational Studies in Epidemiology* (STROBE)^(8)^.

### Diseño del estudio y lugar

Estudio de corte transversal y analítico, realizado con adultos mayores en un hospital de mediana complejidad, de referencia para adultos mayores, ubicado en la región Sur de Brasil.

### Población, participantes, criterios de inclusión y exclusión

La población del estudio estuvo constituida por adultos mayores hospitalizados en la enfermería. Se incluyeron adultos mayores con 60 años o más, hospitalizados en enfermerías clínicas o quirúrgicas, con demanda de cuidados evaluada durante la hospitalización y con capacidad cognitiva monitoreada por el Miniexamen del Estado Mental (MMSE)^(9)^, considerando el nivel de educación para los puntos de corte^(10)^. Se excluyeron los adultos mayores con indicación de internación en Unidad de Cuidados Intensivos (UCI) y bajo precaución de gotículas. Se incluyeron los acompañantes con 18 años o más, que acompañaran al adulto mayor hace al menos tres meses; para los acompañantes con 60 años o más, se realizó la prueba de rastreo cognitivo mediante el MEEM^(9)^, con puntos de corte adoptados según la escolaridad^(10)^.

### Cálculo de la muestra y recopilación de datos

Se utilizó el cálculo de la muestra para la población ≥ 60 años, representativa de las hospitalizaciones en el año 2019 (n=4.146). El nivel de confianza se estableció en el 95% con un error de muestreo del 5%. Considerando los valores para cada parámetro, se obtuvo un tamaño de muestra mínimo de 350 individuos. En este estudio, se adoptó el tamaño de la muestra de 400 participantes para lograr un error de muestreo inferior al 5%.

Previamente a la recopilación de datos, con el objetivo de estandarizar la ejecución y minimizar los sesgos de recopilación, se realizaron capacitaciones teóricas y prácticas con el equipo de examinadores integrantes del grupo de investigación. El equipo estuvo formado por discentes de grado y posgrado de una institución federal de educación superior. Los datos se obtuvieron de marzo del 2022 a marzo del 2023. El tiempo promedio de aplicación de los instrumentos de recopilación fue de aproximadamente 30 minutos.

Inicialmente, el rastreo cognitivo se realizó utilizando el MEEM^(9)^, validado para portugués de Brasil, y se definieron diferentes puntos de corte según el nivel de educación de los participantes. En el caso de las personas analfabetas, el punto de corte se estableció en 13 puntos o menos. En el caso de las personas con uno a ocho años incompletos de escolaridad, se adoptó el punto de corte de 18 puntos o menos. En el caso de los participantes con ocho o más años de escolaridad, el puntaje límite se estableció en 26 puntos o menos^(10)^. Se excluyeron los participantes cuyos puntajes en el MEEM quedaron por debajo de los puntos de corte específicos para su nivel de educación, ya que indicaban un posible déficit cognitivo, con el objetivo de seleccionar adultos mayores capaces de responder los cuestionarios del estudio y firmar el Consentimiento Libre, Previo e Informado (CLPI).

Cuando el adulto mayor presentó alteración cognitiva, se invitó al acompañante a responder las preguntas en presencia del adulto mayor y firmar el CLPI. En los casos en que el acompañante, presente en el momento de la entrevista, no se sintió seguro para responder las preguntas, el equipo esperó a que lo sustituyera el acompañante principal, con quien se reanudó el contacto en persona en una visita posterior. Dicho procedimiento tuvo como objetivo asegurar la confiabilidad de los datos. A continuación el acompañante entrevistado firmaba el CLPI.

La recopilación consistió en la aplicación de un cuestionario sociodemográfico que incluyó: sexo, edad, color de piel, escolaridad e ingreso familiar. Las pruebas recomendadas por el fenotipo de fragilidad física se aplicaron a los siguientes componentes: FPM y VM, fatiga/agotamiento, pérdida de peso no intencionada y nivel de actividad física. Siguiendo los criterios de Fried, se consideró frágil el adulto mayor que presentaba tres o más marcadores; prefrágil, uno o dos; y no frágil, ninguno^(3)^.

La FPM medida por medio de un dinamómetro hidráulico Jamar en kilogramos/fuerza (Kgf). Se le indicó al adulto mayor que permaneciera sentado en una silla con los pies apoyados en el suelo, el codo flexionado a 90 grados, el brazo firmemente contra el torso y la muñeca en una posición neutral. El agarre de la mano dominante se ajustó al dinamómetro de tal manera que la segunda falange del segundo, tercer y cuarto dedo tocaran la curva del eje del dispositivo. Al recibir la orden, el individuo examinado realizó tres agarres, intercalados durante un minuto para recuperar la fuerza, y se consideró el promedio de las tres mediciones. Los valores de la FPM se ajustaron según el cuartil del Índice de Masa Corporal (IMC) y el sexo, y aquellos que comprendían el quintil más bajo de fuerza fueron considerados frágiles para este marcador^(3)^.

Para evaluar la VM, se le indicó al adulto mayor que caminara, de su manera habitual, sobre una superficie plana, marcada con una cinta métrica de 6,6 metros. Los participantes que dependían de dispositivos de ayuda para la movilidad o para caminar realizaron la prueba utilizando estos equipos. Cuando el adulto mayor estaba postrado en cama, se atribuyó el valor cero a este componente.

El marcador de fatiga/agotamiento se identificó mediante autoinforme. Se preguntó a los adultos mayores sobre la frecuencia de las siguientes situaciones durante la última semana: (A) sintió que tenía que hacer un esfuerzo para completar sus tareas diarias y (B) sintió que era incapaz de hacer las cosas. Las respuestas se clasificaron como 0 – rara vez o ninguna parte del tiempo (<1 día); 1 – una parte o pequeña parte del tiempo (1 a 2 días); 2 – cantidad moderada de tiempo (3 a 4 días); o 3 – la mayor parte del tiempo. Una respuesta de “2” o “3” a cualquiera de las preguntas categorizó al adulto mayor como frágil para este componente^(3)^.

Para evaluar la pérdida de peso no intencionada se utilizó una báscula de plataforma digital y un estadiómetro. Se midió a través del IMC, calculado a partir de medidas antropométricas, asociada al autoinforme del adulto mayor en respuesta a dos preguntas: (A) “¿Ha perdido peso en los últimos meses?” y (B) “¿Cuántos kilos?”.

El nivel de actividad física se evaluó mediante el *Minnesotta Leisure Activity Questionnaire*, validado para adultos mayores brasileños^(11)^. Se hicieron preguntas sobre la frecuencia y duración de las actividades realizadas en las últimas dos semanas. Para el valor en kilocalorías, se multiplicó la intensidad de cada actividad en *Metabolic Equivalent Tasks* por la constante 0,0175 y el peso del individuo en kilogramos, y el valor total se dividió entre dos para obtener el gasto energético promedio por semana. Siguiendo el criterio de Fried^(3)^, luego de ajustar por sexo, el adulto mayor que presentó valores en el quintil más bajo se clasificó como frágil para este marcador.

La demanda de cuidados se evaluó mediante la escala de Fugulin^(6)^, que analiza 12 dimensiones del cuidado: estado mental, oxigenación, signos vitales, motilidad, deambulación, alimentación, cuidado corporal, eliminación, terapéutica, integridad cutánea/mucosa/deterioro tisular, apósitos, tiempo empleado para cambiar los apósitos. Cada dimensión presenta cuatro indicadores, que reciben un puntaje de uno a cuatro, según la dependencia de la asistencia de enfermería. La graduación final de la escala Fugulin varía de 12 a 48 puntos, mientras que, cuanto mayor sea el puntaje, mayor será la necesidad de asistencia y cuidados de enfermería. Los puntajes de 12 a 17 se consideraron cuidados mínimos, los de 18 a 22 puntos, cuidados intermedios; los de 23 a 28 puntos, cuidados de alta dependencia; los de 29 a 34 puntos, cuidados semiintensivos, y los puntajes por encima de 34 se consideraron cuidados intensivos^(6)^.

### Análisis y tratamiento de los datos

Se construyó una base de datos utilizando hojas de cálculo de *Microsoft Excel 2016*, y los datos se ingresaron con doble comprobación. Para el análisis descriptivo, se utilizaron medias, medianas, tablas de frecuencia y proporciones con sus respectivos intervalos de confianza (IC) del 95%. Las comparaciones entre los grupos de adultos mayores frágiles, prefrágiles y no frágiles^(3)^ se realizaron por medio de la prueba exacta de Fisher, para variables categóricas. Para las variables numéricas y categóricas, la comparación se realizó mediante la prueba de Kruskal-Wallis, seguida de la prueba de Dunn para comparaciones múltiples. En los análisis estadísticos, se consideró el nivel de significancia de *p* ≤0,05.

### Aspectos éticos

El estudio fue aprobado por el Comité de Ética en Investigación en Seres Humanos del Sector de Ciencias de la Salud de la Universidade Federal do Paraná y por el Comité de Ética en Investigación en Seres Humanos de la Secretaría Municipal de Salud, dictamen n.º 5.055.260. La firma del CLPI por parte del adulto mayor o acompañante se realizó antes del inicio de la investigación, de acuerdo con la Resolución n.º 466/2012. Asimismo, se respetó el secreto y la confidencialidad de los datos, en los términos expresados en las Resoluciones n.º 510/2016, n.º 580/2018 y demás resoluciones del Consejo Nacional de Salud vigentes a la fecha de este estudio.

## Resultados

En la Tabla 1, se presentan los resultados respecto a las características sociodemográficas de los 400 participantes del estudio. De estos, el 55,8% (n=223) son mujeres, el 70,85% (n=283) se autodeclara blanco, el 36,5% (n=146) tiene más de 80 años, el 51,5% (n=206) no completó la educación primaria y el 39,8% (n=159) recibe entre 1 y 3 salarios mínimos.

**Tabla 1-:** Distribución de frecuencias de las características sociodemográficas de los adultos mayores de la muestra. Curitiba, PR, Brasil, 2024

**Características**	n*=400	**(IC** ^†^ **95%)**
Sexo		
Femenino	223	55,8% (50,7%-60,7%)
Masculino	177	44,3% (39,3%-49,3%)
Edad		
60 a 69 años	118	29,5% (25,1%-34,3%)
70 a 79 años	136	34% (29,4%-38,9%)
80 años o más	146	36,5% (31,8%-41,5%)
Color de la piel		
Amarilla	3	0,8% (0,19%-2,36%)
Blanca	283	70,8% (66,0%-75,1%)
Parda	90	22,6% (18,4%-28,31%)
Negra	24	6% (3,96%-8,92%)
Escolaridad		
Analfabeto/a	72	18% (14,4%-22,2%)
Alfabetizado/a	38	9,5% (6,89%-12,9%)
Educación primaria incompleta	206	51,5% (46,5%-56,5%)
Educación primaria	22	5,5% (3,56%-8,33%)
Educación secundaria incompleta	12	3% (1,63%-5,33%)
Educación secundaria	28	7% (4,78%-10,1%)
Educación superior incompleta	4	1% (0,32%-2,72%)
Educación superior	18	4,5% (2,77%-7,15%)
Ingreso familiar (MW ^‡^ )		
Sin ingresos	22	5,5% (3,56%-8,33%)
0-1	135	33,8% (29,2%-38,6%)
1,1-3	159	39,8% (35%-44,7%)
3,1-5	55	13,8% (10,6%-17,6%)
5,1-10	5	1,3% (0,46%-3,6%)
Mayor que 10	1	0,3% (0,01%-1,61%)
No quiere informar	23	5,8% (3,76%-8,63%)

^*^n = Número de participantes; ^†^IC = Intervalo de confianza; ^‡^SM = Salario mínimo en el año 2024: R$ 1.412,00^(12)^

En la Tabla 2, se observa el predominio de adultos mayores prefrágiles, el 48,7% (n=195); los frágiles alcanzaron el 35,8% (n=143); y los no frágiles totalizaron el 15,5% (n=62). Respecto a la necesidad de cuidados de alta dependencia, los porcentajes para los adultos mayores frágiles, prefrágiles y no frágiles fueron los siguientes, respectivamente: 44,8% (n=64), 10,8% (n=21) y 3,2% (n=2), *p*<0,001. Se encontró una diferencia estadísticamente significativa entre los grupos en cuanto a la demanda de cuidados mínimos (*p*<0,001), lo que indica que la proporción de adultos mayores que requieren cuidados mínimos varía significativamente dependiendo de su fragilidad física. La demanda de cuidados mínimos se observó en el 80,6% de los no frágiles, el 60,5% de los prefrágiles y el 14% de los frágiles, lo que sugiere que la reducción de la fragilidad física está asociada con una menor demanda de cuidados más complejos.

**Tabla 2-:** Asociación entre la fragilidad física y la demanda de cuidados de adultos mayores de la muestra. Curitiba, PR, Brasil, 2024

**Categorización**	n*=400	**Clasificación de la fragilidad**			**Valor *p*** ^†^
		**No frágil** n=62	**Prefrágil** n=195	**Frágil** n=143	
**Cuidados mínimos**	**188** **47%** **(42%-52%)**	**80,6%** **(68.3%-89.2%)**	**60,5%** **(53,3%-67,4%)**	**14%** **(8,96%-21%)**	**<0,001**
Cuidados intermedios	89 22,3% (18,3%-26,7%)	16,1% (8,41%-28,1%)	27,7% (21,7%-34,6%)	17,5% (11,8%-24,9%)	
**Cuidados de alta dependencia**	**87** 21,6% (17,9%-26,2%)	3,2% (0,56%-12,2%)	10,8% (6,94%-16,2%)	**44,8%** **(36,5%-53,3%)**	
Cuidados semiintensivos	29 7,3% (4,99%-10,4%)	0% (0%-7,27%)	1% (0,18%-4,05%)	18,8% (13%-26,5%)	
Cuidados intensivos	7 1,8% (0,77%-3,73%)	0% (0%-7,27%)	0% (0%-2,41%)	4,9% (2,16%-10,2%)	

^*^n = Número de participantes; ^†^*p* = Probabilidad de significancia por la prueba exacta de Fisher

En la Tabla 3 se muestra la asociación del puntaje de cada área del cuidado con las diferentes condiciones de fragilidad. Los adultos mayores clasificados como frágiles (n=143) requirieron asistencia de enfermería superior que los no frágiles en las siguientes áreas de cuidado: estado mental, oxigenación, motilidad, deambulación, eliminación y terapéutica (*p<*0,001). En comparación con los individuos prefrágiles, los frágiles obtuvieron puntajes más altos en todas las áreas del cuidado, deterioro tisular (*p=*0,031), apósitos (*p=*0,004) y el tiempo dedicado a cambiar los apósitos (*p<*0,001). Los pacientes prefrágiles obtuvieron puntajes más altos que los pacientes no frágiles en las áreas de deambulación (*p<*0,001), cuidado corporal (*p<*0,001) y eliminación (*p<*0,001). Al comparar los puntajes totales de Fugulin^(6)^, entre los tres grupos de pacientes, todos los grupos eran diferentes entre sí, y los puntajes medios eran más altos a medida que aumentaba la fragilidad (*p=*0,001).

**Tabla 3-:** Asociación entre el puntaje medio de las áreas de cuidado y la fragilidad física de los adultos mayores de la muestra. Curitiba, PR, Brasil, 2024

Área del cuidado	**Clasificación de Fried**			**Valor *p*** ^†^	**Diferencias** **significativas** ^‡^
	**No frágil** n*=62	**Prefrágil** n*=195	**Frágil** n*=143		
Estado mental	1 (1-1,1)	1,1 (1,1-1,2)	1,8 (1,7-2)	<0,001	Frágil – No frágil, Frágil – Prefrágil
Oxigenación	1,1 (1-1,2)	1,2 (1,2-1,3)	1,6 (1,5-1,8)	<0,001	Frágil – No frágil, Frágil – Prefrágil
Signos vitales	2	2	2	0,298	
Motilidad	1,2 (1,1-1,3)	1,4 (1,3-1,5)	2,4 (2,2-2,6)	<0,001	Frágil – No frágil, Frágil – Prefrágil
Deambulación	1,1 (1-1,3)	1,6 (1,5-1,8)	3 (2,8-3,2)	<0,001	Frágil – No Frágil, Frágil – Prefrágil, No frágil – Prefrágil
Alimentación	1 (1-1)	1,1 (1-1,1)	1,6 (1,5-1,8)	<0,001	Frágil – No frágil, Frágil – Prefrágil
Cuidado corporal	1,3 (1,1-1,4)	1,8 (1,6-2)	3,2 (3-3,4)	<0,001	Frágil – No Frágil, Frágil – Prefrágil, No frágil – Prefrágil
Eliminación	1,4 (1,2-1,6)	1,8 (1,6-1,9)	2,8 (2,7-3)	<0,001	Frágil – No Frágil, Frágil – Prefrágil, No frágil – Prefrágil
Terapéutica	2 (2-2)	2 (2-2)	2,1 (2-2,2)	<0,001	Frágil – No frágil, Frágil – Prefrágil
Deterioro tisular	1,3 (1,1-1,4)	1,2 (1,1-1,3)	1,3 (1,2-1,5)	0,031	Frágil – Prefrágil
Apósitos	1,1 (1-1,2)	1,1 (1-1,1)	1,2 (1,1-1,3)	0,004	Frágil – Prefrágil
Tiempo para cambiar los apósitos	1,1 (1-1,2)	1 (1-1,1)	1,2 (1,1-1,3)	<0,001	Frágil – Prefrágil
Puntaje total	15,6 (15,0-16,3)	17,3 (16,8-17,8)	24,4 (23,5-25,4)	<0,001	Frágil – No Frágil, Frágil – Prefrágil, No frágil – Prefrágil

*n = Número de participantes; ^†^*p* = Probabilidad de significancia por la prueba de Kruskal-Wallis; ^‡^Prueba de Dunn

Se encontró que los adultos mayores en cuidados intensivos presentaron una reducción de la FPM, de la VM y fatiga/agotamiento autoinformados (100%), y el 85,7% presentó una disminución en el nivel de actividad física y pérdida de peso no intencional. En aquellos en cuidados mínimos, el 7,4% se identificó con FPM reducido, el 3,2% con velocidad de marcha reducida, el 25,5% tuvo un nivel disminuido de actividad física, el 37,8% tuvo pérdida de peso no intencional y el 43,1% reportó fatiga/agotamiento. Se observó una diferencia estadísticamente significativa entre los grupos con relación a todos los marcadores del fenotipo fragilidad, como lo indican los valores de *p*, lo que pone de manifiesto una asociación entre una mayor necesidad de cuidados y un peor rendimiento en los marcadores de fragilidad (Tabla 4).

**Tabla 4-:** Asociación entre la demanda de cuidados y los marcadores del fenotipo de fragilidad de los adultos mayores de la muestra. Curitiba, PR, Brasil, 2024

**Marcadores**	n*=400	**Nivel de cuidados**					**Valor *p*** ^†^
		**Mínimos** n*=188	**Intermedios** n*=89	**Alta dependencia** n*=87	**Semiintensivos** n*=29	**Intensivos** n*=7	
Reducción de la fuerza de agarre de la mano	23%	7,4% (4,29%-12,4%)	14,6% (8,31%-24%)	40,2% (30%-51,3%)	79,3% (59,7%-91,3%)	100% (56,1%-100%)	**<0,001**
Reducción de la velocidad al caminar	41%	3,2% (1,3%-7,14%)	41,6% (31,4%-52,5%)	96,6% (89,5%-99,1%)	100% (85,4%-100%)	100% (56,1%-100%)	**<0,001**
Disminución del nivel de actividad física	44%	25,5% (19,6%-32,5%)	41,6% (31,4%-52,5%)	71,3% (60,4%-80,2%)	82,8% (63,5%-93,5%)	85,7% (42%-99,2%)	**<0,001**
Pérdida de peso no intencionada	55%	37,8% (30,9%-45,1%)	40,4% (30,3%-51,4%)	49,4% (38,6%-60,3%)	58,6% (39,1%-75,9%)	85,7% (42%-99,2%)	**0,019**
Fatiga/agotamiento autoinformados	43%	43,1% (36%-50,5%)	55,1% (44, 2%-65,5%)	65,5% (54,5%-75,2%)	82,8% (63,5%-93,5%)	100% (56,1%-100%)	**<0,001**

*n = Número de participantes; ^†^*p* = Probabilidad de significancia por la prueba exacta de Fisher

## Discusión

De los 400 participantes del estudio, predominaron las mujeres, de piel blanca, con 80 años o más, escolaridad primaria incompleta y con ingreso familiar de 1 a 3 salarios mínimos. El número de mujeres en el ámbito hospitalario refleja el proceso de feminización del envejecimiento, lo que representa el 51,5% de la población anciana del país^(13)^.

En este estudio, la representatividad de los octogenarios fue del 36,5%. Datos similares se encontraron en un estudio realizado en São Paulo (Brasil), con 865 adultos mayores hospitalizados, que identificó el 53,9% de mujeres y el 64,6% de personas longevas^(14)^. La mayoría de los participantes tenía niveles bajos de educación (educación primaria incompleta), en particular un 18% era analfabeto. Este porcentaje es similar a la tasa nacional registrada en Brasil (16%)^(13)^.

El predominio de individuos prefrágiles (48,8%) seguido de frágiles (35,8%) contrasta con lo identificado en una revisión sistemática, que tuvo como objetivo identificar la prevalencia de fragilidad en 467.779 adultos mayores hospitalizados, de 65 años o más. Los resultados mostraron un 47% de frágiles y un 26% de prefrágiles. Sin embargo, los autores señalan la imprecisión en la definición operativa de fragilidad en la investigación como un punto que discutir^(15)^.

En este estudio, alrededor del 50% de los adultos mayores requirió cuidados mínimos y alrededor del 20% requirió cuidados intermedios y de alta dependencia. Un estudio retrospectivo evaluó 161 registros médicos de adultos mayores en un hospital universitario de Maranhão (Brasil). De la muestra, el 63,4% requirió cuidados mínimos, el 15% cuidados intermedios, el 14,2% de alta dependencia, el 5,6% cuidados semiintensivos y el 1,8% cuidados intensivos^(7)^.

Otro estudio nacional, realizado en un servicio de urgencias de un hospital, tuvo como objetivo clasificar el grado de dependencia de los ingresados. De los 783 individuos evaluados, el 40,8% tenía 60 años o más. Se observó que el 37% necesitó cuidados mínimos, el 31,7% apoyo intermedio, el 22,8% cuidados de alta dependencia, el 7,4% cuidados semiintensivos y el 1,3% cuidados intensivos^(16)^. Al comparar estos resultados con los de este estudio, se encuentra la misma secuencia de clasificación, con una disminución del 10% en los cuidados mínimos y un aumento del 10% en los cuidados intermedios. Estas diferencias pueden atribuirse a variaciones en el perfil de los adultos mayores, así como a las diferentes características de los entornos hospitalarios.

La mayor demanda de cuidados de enfermería se asocia a una mayor fragilidad. De los individuos frágiles, respectivamente, los resultados para quienes recibieron cuidados de alta dependencia, semiintensivos e intensivos fueron los siguientes: 44,8%, 18,9% y 4,9%. Del grupo de personas prefrágiles, el 60,5% recibió cuidados mínimos; el 27,7% cuidados intermedios; y el 10,8% cuidados de alta dependencia. A su vez, de los no frágiles, el 80,6% requirió cuidados mínimos y el 16,1% cuidados intermedios. Se constató una brecha significativa en los estudios en la literatura nacional e internacional respecto a la relación entre los niveles de cuidados de enfermería y la condición de fragilidad física en adultos mayores hospitalizados. Por lo tanto, se destaca el estudio transversal que se llevó a cabo en una Institución de Cuidados de Larga Duración (ICLD) en China. Los datos indicaron una asociación entre la fragilidad y las necesidades de cuidados (OR 3,06, IC 95%; 2,06-4,55, *p*<0,01). Según los investigadores, existe una relación lineal entre la fragilidad y las necesidades de cuidados. A medida que aumenta el grado de fragilidad, aumentan drásticamente las necesidades de cuidados de larga duración de los adultos mayores^(17)^.

Se identificaron diferencias entre adultos mayores en condiciones no frágiles, prefrágiles y frágiles en todas las áreas evaluadas. Los adultos mayores frágiles requieren mayores cuidados e intervenciones de enfermería en comparación con los individuos prefrágiles, expresados en las áreas de estado mental, oxigenación, motilidad, deambulación, alimentación, cuidado corporal, eliminación, terapéutica y deterioro tisular. En los adultos mayores frágiles, las discapacidades más comunes fueron la motilidad (movimiento con ayuda o incapacidad para moverse), la deambulación (restringidos a la cama o postrados), el cuidado corporal (baño en cama e higiene corporal realizada por el equipo de enfermería), la eliminación (uso de bacinilla o eliminación en la cama). Estas discapacidades se consideraron las más limitantes y las que expresaron mayor necesidad de cuidados de enfermería (*p*<0,001). La fragilidad física puede considerarse un factor preincapacitante^(2)^, que dificulta la realización de actividades básicas de la vida diaria, está asociada al deterioro cognitivo, al aumento de la propensión a caídas^(18)^, además de aumentar la susceptibilidad al riesgo de que el adulto mayor sufra violencia^(19)^.

Respecto a los marcadores del fenotipo de fragilidad física, se observó un aumento de la prevalencia a medida que crecía la demanda de cuidados. Los marcadores de reducción de VM y FPM, fatiga/agotamiento autoinformados se identificaron en el 100% de los adultos mayores con necesidades de cuidados intensivos. Se encontró asociación entre los cuidados que requerían y los marcadores del fenotipo de fragilidad física.

El síndrome de fragilidad es un precursor fisiológico de la incapacidad, caracterizada por debilidad, disminución de la resistencia y rendimiento lento. Estos aspectos centrales de la fragilidad afectan, principalmente, a funciones dependientes de la energía y la velocidad, como la movilidad^(3)^. Un estudio con adultos mayores hospitalizados identificó la fragilidad como un factor predictivo de disminución de la movilidad^(20)^. Las intervenciones específicas pueden interrumpir, ralentizar o revertir este declive^(2)^. Los resultados de este estudio indican que, con el aumento de la necesidad de cuidados — desde mínimos a intensivos—, se observa un incremento proporcional de la debilidad muscular, evidenciada por mayores limitaciones funcionales.

El predominio de marcadores de fragilidad física, como la reducción de la VM, la reducción de la FPM y fatiga/agotamiento autoinformados, en adultos mayores que requirieron cuidados intensivos, indica una pérdida sustancial de fuerza muscular y resistencia, identificada por las áreas de necesidad de cuidados. Estos marcadores son indicativos de una probable sarcopenia, que es la pérdida progresiva y generalizada de masa y fuerza o función muscular^(21)^. La sarcopenia y la fragilidad física son afecciones prevalentes en adultos mayores hospitalizados^(22)^.

La fatiga/agotamiento autoinformados fue el marcador más frecuente entre los adultos mayores que recibían cuidados mínimos e intermedios. Así, la referencia por parte de los adultos mayores a este marcador aislado anticipa la aparición de fragilidad física en ellos. En estudios, a menudo se muestra predominante en comparación con los otros marcadores. Los datos de un estudio transversal desarrollado en un hospital de Irán indicaron que el marcador de agotamiento estaba presente en el 83% de los adultos mayores hospitalizados^(23)^. En otro estudio transversal, los adultos mayores prefrágiles sin signos clínicos de agotamiento experimentaron una menor fuerza de agarre, una mayor fatiga autopercibida y niveles más bajos de vitalidad en comparación con los robustos (*p*<0,001)^(24)^.

Otro marcador con porcentajes significativos fue la pérdida de peso no intencionada, autoinformada en el 43% de los ancianos hospitalizados. Los expertos en fragilidad física recomiendan a los profesionales de la salud que identifiquen las causas tratables de la pérdida de peso^(2)^. Las personas con pérdida de peso no intencionada (≥4,5 kg en un año) y las personas con pérdida no intencionada del 25% del peso corporal, a lo largo de un año, se consideran población en riesgo para el diagnóstico de enfermería: “riesgo de” o “síndrome de fragilidad del adulto mayor”^(25)^. En el proceso de pérdida de peso, no solo se produce una reducción del tejido adiposo, sino también una disminución acelerada de la masa magra, y este es uno de los principales factores para el desarrollo de la sarcopenia^(21)^. Cabe señalar que esta variable corresponde a información autorreferida, cuya precisión depende de la preservación de las funciones cognitivas y del nivel de consciencia de los adultos mayores.

La reducción de la VM afectó al 41% de la muestra y se observó en el 96,6% de los individuos con necesidades de cuidados de alta dependencia y en el 100% de los que estaban en cuidados semiintensivos y con un porcentaje menos expresivo en cuidados mínimos (3,2%). La disminución de la velocidad de la marcha afecta áreas de cuidado como: motilidad, deambulación, cuidado corporal y eliminación^(26)^. Los adultos mayores frágiles presentan un empeoramiento de la marcha y del rendimiento en tareas simultáneas (motoras y cognitivas)^(27)^.

La reducción del marcador de fragilidad FPM fue la que alcanzó el menor porcentaje (23%), sin embargo, estuvo presente en todos (100%) los adultos mayores en cuidados intensivos. La manifestación de este marcador parece estar relacionada con el estímulo que proporcionan las actividades manuales que aún realizan los adultos mayores, incluso aquellos que están postrados en cama. Esto corrobora el porcentaje encontrado en el estudio realizado en España, que totalizó una reducción del 20,4% en la FPM, y este marcador se asoció a una menor probabilidad de recuperar la no fragilidad. Los autores añaden que este marcador de fragilidad es, a menudo, el último en manifestarse^(28)^. El uso más frecuente de los miembros superiores en las actividades diarias mantiene la fuerza y la resistencia muscular de estos miembros en adultos mayores.

La condición de fragilidad física identificada en la muestra expresa la necesidad de cuidados de enfermería además de cuidados de las morbilidades que llevaron a la hospitalización. En este complemento a los cuidados, se destaca la importancia de la identificación precoz de marcadores y la necesidad de incorporar la valoración de la fragilidad al ingreso hospitalario^(29)^. Algunos estudios identificaron la necesidad de mejorar las habilidades de los enfermeros para reconocer y manejar la condición de fragilidad^(30-31)^, una vez que son los profesionales que están en contacto directo y frecuente con el adulto mayor hospitalizado^(32)^.

Este enfoque permite dirigir las intervenciones de enfermería orientadas a promover el autocuidado, con énfasis en el fortalecimiento de las habilidades funcionales, en lugar de centrarse en las limitaciones de los individuos.

Este estudio demuestra que la fragilidad física compromete la movilidad, la eliminación, el cuidado corporal y la adhesión terapéutica. Se resalta la importancia de identificar precozmente esta condición en adultos mayores hospitalizados para apoyar intervenciones específicas, optimizar el dimensionamiento del equipo y calificar el plan de cuidados, con el objetivo de ralentizar, interrumpir o revertir la progresión de la fragilidad en el contexto hospitalario. La educación en salud dirigida a los adultos mayores y sus familias, combinada con el fortalecimiento del apoyo familiar y la promoción del conocimiento acerca de la condición clínica y la adhesión al tratamiento, es una estrategia relevante en el contexto del cuidado de enfermería.

La identificación de la fragilidad se considera como un cuidado inicial que orienta los cuidados que se deben establecer, con el objetivo de evitar los efectos negativos de la transición de la fragilidad a condiciones avanzadas, durante el período de hospitalización. Para ello, es necesario que el equipo de enfermería y el equipo multiprofesional desarrollen planes de cuidados integrados, basados en la práctica de ejercicios físicos de fuerza, resistencia y aeróbicos, nutrición con soporte proteico y calórico, prescripción de suplementación con vitamina D y reducción de la polifarmacia^(2)^.

Este estudio contribuye a la enfermería geriátrica al demostrar la asociación entre los marcadores de fragilidad (reducción de la fuerza de agarre, disminución de la velocidad de marcha y fatiga) y una mayor dependencia funcional durante la hospitalización. La identificación temprana de estos marcadores por parte del equipo de enfermería permite intervenciones individualizadas, lo que favorece el mantenimiento de la funcionalidad y previene resultados adversos, como inmovilidad, delirio, caídas y pérdida de autonomía.

Asimismo, el estudio apoya la práctica clínica de enfermería geriátrica al proponer la incorporación de la evaluación de la fragilidad física como herramienta sistemática al momento del ingreso hospitalario. Este enfoque apoya la planificación del cuidado con base en evidencias y contribuye al dimensionamiento adecuado del equipo, la asignación racional de recursos y el desarrollo de planes terapéuticos más efectivos. Al reconocer la fragilidad como una condición potencialmente reversible, esta investigación también refuerza la importancia de estrategias de cuidado centradas en la rehabilitación funcional, la educación para la salud y la promoción de la autonomía, alineadas con los principios del envejecimiento activo y humanización de la atención hospitalaria a los adultos mayores.

Como limitación, se destaca el uso del instrumento SCP, que requiere evaluaciones más integrales, que incluyan aspectos sociales y psicológicos, además de la escasez de estudios que relacionan la fragilidad física y la demanda de cuidados, lo que restringió la discusión de los resultados. El uso de datos autoinformados, como fatiga, agotamiento y pérdida de peso, también constituye una limitación, aunque este sesgo ha sido minimizado mediante el rigor metodológico, la capacitación de los entrevistadores, la calibración de los instrumentos y el apoyo estadístico. El sesgo de observación se redujo mediante el desarrollo de un protocolo estandarizado de recopilación y análisis. Como punto positivo, se resalta la representatividad de la muestra, proveniente de un hospital vinculado al Sistema Único de Salud (SUS).

## Conclusión

La condición de fragilidad física está asociada a una mayor demanda de cuidados de enfermería en adultos mayores hospitalizados. Se constató que los adultos mayores frágiles presentaron una mayor necesidad de cuidados de alta dependencia, mientras que los prefrágiles y no frágiles requerían predominantemente cuidados mínimos. Las áreas de mayor deterioro entre los adultos mayores frágiles hospitalizados fueron la deambulación, el cuidado corporal y la eliminación, lo que pone de manifiesto limitaciones funcionales que requieren intervenciones específicas del equipo de enfermería.

En este contexto, se resalta la relevancia de evaluar sistemáticamente la fragilidad física al ingreso hospitalario, centrándose en marcadores clínicos como la reducción de la velocidad de marcha y de la fuerza de agarre manual y la fatiga/agotamiento autoinformados. La identificación temprana de estos indicadores puede apoyar la planificación individualizada de la asistencia, optimizar el dimensionamiento de la fuerza de trabajo y favorecer la implementación de estrategias dirigidas al mantenimiento o recuperación de la funcionalidad, lo que contribuye a la cualificación de la atención.

## Data Availability

Los conjuntos de datos relacionados con este artículo estarán disponibles previa solicitud al autor correspondiente.
